# Efficacy and safety of AAV-mediated gene therapy for choroideremia: a systematic review and meta-analysis

**DOI:** 10.1016/j.eclinm.2026.103923

**Published:** 2026-05-11

**Authors:** Kai-Yang Chen, Hoi-Chun Chan, Chi-Ming Chan

**Affiliations:** a Department of General Medicine, Chang Gung Memorial Hospital (Linkou branch), Taoyuan, Taiwan; b School of Pharmacy, China Medical University, Taichung, Taiwan; c Department of Ophthalmology, Cardinal Tien Hospital, New Taipei City, Taiwan; d School of Medicine, Fu Jen Catholic University, New Taipei City, Taiwan

**Keywords:** AAV2-mediated gene therapy, Choroideremia, *CHM* gene, REP1, Microperimetry, Fundus autofluorescence

## Abstract

**Background:**

Choroideremia is a rare X-linked inherited retinal dystrophy caused by mutations in the *CHM* gene, leading to progressive degeneration of the retinal pigment epithelium (RPE), choroid, and photoreceptors. Current management remains largely supportive, and gene therapy has emerged as a potential disease-modifying treatment. This study aimed to systematically evaluate the efficacy and safety of adeno-associated virus (AAV)–mediated gene therapy for choroideremia.

**Methods:**

A systematic review and meta-analysis were conducted in accordance with Preferred Reporting Items for Systematic reviews and Meta-Analyses (PRISMA) 2020 guidelines and registered in International Prospective Register of Systematic Reviews (PROSPERO) (CRD420251146173). Six databases (PubMed, Embase, Scopus, ScienceDirect, Web of Science, and the Cochrane Library) were searched from inception to December 3, 2025. Clinical trials and prospective observational studies evaluating AAV-mediated gene therapy targeting the Rab escort protein 1 (REP1) gene were included. Outcomes included best-corrected visual acuity (BCVA), retinal sensitivity measured by microperimetry, preserved RPE area on fundus autofluorescence (FAF), subfoveal choroidal thickness, and treatment-emergent adverse events (TEAEs). Random-effects meta-analyses were performed using Comprehensive Meta-Analysis software.

**Findings:**

Eleven clinical studies involving 308 participants were included. Gene therapy demonstrated a significant improvement in retinal sensitivity (mean difference [MD] 0.78 dB, 95% CI: 0.58–0.99, p < 0.001) with consistent effects across follow-up durations up to 48 months. BCVA showed a significant pooled improvement of 3.07 Early Treatment Diabetic Retinopathy Study (ETDRS) letters (95% CI: 1.85–4.30, p < 0.001), with greater gains observed at 24 months. Structural outcomes indicated reduced RPE degeneration (MD −4.41, 95% CI: −6.39 to −2.44, p < 0.001) and increased subfoveal choroidal thickness (MD 9.13 μm, 95% CI: 7.53–10.72, p < 0.001). TEAEs occurred in approximately 35% of treated participants in the pooled event-rate analysis, with the majority of events being mild to moderate and procedure-related, and a relatively low incidence of serious adverse events (<20%).

**Interpretation:**

AAV-mediated gene therapy for choroideremia demonstrates modest functional benefits and structural preservation signals. TEAEs are relatively common and are predominantly procedure-associated ocular events, although most reported events are mild to moderate and serious complications remain uncommon. The therapy appears to act primarily as a disease-modifying intervention that stabilizes retinal degeneration rather than restoring vision, supporting further trials with earlier intervention, optimized delivery strategies, and longer follow-up.

**Funding:**

No specific funding was received from any funding bodies in the public, commercial, or not-for-profit sectors.

**Research in context**

**Evidence before this study**

Before undertaking this systematic review and meta-analysis, we searched PubMed, Embase, Scopus, ScienceDirect, Web of Science, and the Cochrane Library from database inception to December 3, 2025, without language restrictions. We also manually screened reference lists of eligible articles and relevant reviews. Search terms combined disease-related keywords (“choroideremia,” “choroideraemia,” and “CHM gene”) with intervention-related terms (“gene therapy,” “genetic therapy,” “adeno-associated virus [AAV],” “Rab escort protein 1 [REP1],” “adeno-associated virus serotype 2–REP1 [AAV2-REP1],” and “timrepigene emparvovec”), and, where permitted, outcome-related terms including “visual acuity,” “microperimetry,” and “fundus autofluorescence.” We included clinical trials and prospective observational studies evaluating AAV-mediated gene therapy in genetically confirmed choroideremia and excluded non-human studies, reviews, editorials, conference abstracts without sufficient data, studies lacking choroideremia-specific outcomes, and studies of non-AAV interventions. Existing evidence consisted predominantly of small early-phase interventional studies, with one larger randomized Phase III trial and several non-randomized prospective cohorts. Overall, the available evidence suggested modest functional stabilization, variable structural preservation, and a safety profile largely dominated by procedure-related ocular adverse events. Risk of bias was assessed using the Cochrane Risk of Bias 2 (RoB 2) tool for randomized studies and the Risk Of Bias In Non-randomized Studies of Interventions (ROBINS-I) tool for non-randomized studies, with most randomized studies judged as having some concerns and most non-randomized studies judged as low to moderate risk of bias, particularly due to confounding and outcome measurement limitations. No prior quantitative synthesis comprehensively integrated functional, structural, and safety outcomes across the full AAV-mediated choroideremia literature using pooled meta-analytic estimates.

**Added value of this study**

This study provides, to our knowledge, the most comprehensive quantitative synthesis of AAV-mediated gene therapy for choroideremia to date. By integrating evidence from 11 clinical studies involving 308 participants, it quantifies treatment-associated effects across multiple clinically relevant domains, including best-corrected visual acuity, retinal sensitivity by microperimetry, preserved retinal pigment epithelium area on fundus autofluorescence, subfoveal choroidal thickness, and treatment-emergent adverse events. The analysis demonstrates that AAV-mediated gene therapy is associated with modest but statistically significant improvements in retinal sensitivity and visual acuity, along with structural signals consistent with slower retinal degeneration. It also clarifies that adverse events are relatively common but are predominantly mild to moderate and procedure-associated, while serious ocular complications are comparatively uncommon. In addition, this study addresses heterogeneity in comparator structures across the literature, incorporates risk-of-bias and Grading of Recommendations Assessment, Development and Evaluation (GRADE)-based certainty assessments, and places the findings within the context of disease modification rather than visual restoration. These features substantially strengthen the interpretability of the current evidence base.

**Implications of all the available evidence**

Taken together, the available evidence indicates that AAV-mediated gene therapy for choroideremia should be viewed primarily as a disease-modifying intervention that may preserve retinal structure and stabilize visual function, rather than as a therapy that restores vision dramatically. The modest magnitude of functional gain, combined with structural preservation signals and a largely manageable procedure-related safety profile, supports continued clinical development of this therapeutic approach, particularly in patients treated before advanced foveal degeneration. For clinical practice, these findings support cautious optimism and realistic counseling regarding expected benefits and risks in specialized centers experienced in subretinal gene delivery. For research, the evidence underscores the need for larger adequately powered randomized trials, earlier intervention, harmonized outcome definitions, longer follow-up, improved handling of repeated longitudinal measures, and optimized delivery strategies to better define durability, patient selection, and true clinical significance.

## Introduction

Choroideremia is a rare, X-linked inherited retinal dystrophy.^1^ It affects approximately 1 in 50,000 to 100,000 individuals globally.^2^ Its prevalence is greater in males because it is inherited through the X chromosome.^3^ This progressive disorder results from mutations in the X-linked *CHM* gene,^4^ leading to progressive degeneration of the retinal pigment epithelium (RPE), choroid, and photoreceptors.^5^ The X-linked CHM gene encodes Rab escort protein 1 (REP1),^6^ which is essential for Rab protein prenylation and appropriate intracellular membrane trafficking in retinal cells.^7^

Patients with choroideremia typically present with nyctalopia (night blindness) and progressive peripheral vision loss, particularly during childhood or adolescence.^8^ This condition typically progresses with gradual peripheral retinal degeneration and may eventually lead to severe visual impairment later in life, although some patients retain useful central vision into their fifth or sixth decade.^9^ These patients usually suffer from severe vision problems and an inability to maintain independence during basic daily tasks.^10^ Currently, its management remains limited to supportive measures such as low-vision aids, orientation and mobility training, and genetic counseling.^11^ These interventions do not address the underlying pathophysiology or influence disease progression.

Gene therapy has emerged as a promising therapeutic approach for inherited retinal diseases, particularly those caused by single-gene mutations, such as choroideremia.^12^^,^^13^ The eye has an immune-privileged status, a relatively small size, compartmentalization, and accessibility for direct drug delivery and monitoring of therapeutic outcomes, making it a suitable target organ for gene therapy.^14^^,^^15^ Adeno-associated virus serotype 2 (AAV2) vectors exhibit strong tropism for retinal cells, particularly photoreceptors and RPE cells.^16^ AAV2 has low pathogenicity in humans, a generally low immunogenic profile, and the capacity to support sustained transgene expression.^17^ Additionally, it does not integrate into the host genome in a site-specific manner, thereby minimizing the risk of insertional mutagenesis.^18^

AAV2-mediated gene therapy in choroideremia involves the subretinal delivery of a functional copy of the *CHM* gene to restore REP1 protein production in affected retinal cells.^19^ By replacing the defective gene, it potentially halts or slows the progressive degeneration of retinal structures, thereby preserving visual function.^20^

Several Phase I and II clinical trials have evaluated the safety and efficacy of AAV2-mediated gene therapy for choroideremia over the past decade. These pioneering studies have reported various outcome measures, including changes in visual acuity, retinal sensitivity, retinal structure, and safety parameters such as intraocular inflammation, cataract formation, and systemic adverse events.^21^ Given the progressive nature of choroideremia and the limited therapeutic options currently available,^22^ there is a need to synthesize the existing evidence of the efficacy and safety of AAV2-mediated gene therapy in managing choroideremia. Therefore, this comprehensive synthesis will provide insights into the treatment effects and safety outcomes.

The primary objective of this study was to critically evaluate the empirical evidence on the safety and efficacy of AAV2-mediated gene therapy for choroideremia.

## Methods

### Protocol and registration

The protocol for this study was registered with the International Prospective Register of Systematic Reviews (PROSPERO) under the registration number (CRD420251146173). The planning, execution, and reporting of this study adhered to the Preferred Reporting Items for Systematic reviews and Meta-Analyses (PRISMA) statement.^23^ Because this review analyzed only previously published aggregate data, institutional review board approval and informed consent were not required. This review exclusively analyzed secondary data from existing studies and, therefore, qualifies for exemption from informed consent or institutional review board approval.

### Data sources

A comprehensive and systematic literature search was conducted to identify relevant peer-reviewed original studies. The search was performed independently by two reviewers to ensure methodological rigor and completeness. Two reviewers (K. Y. C. and H. C. C.) independently conducted an exhaustive search across six major electronic databases, including PubMed, Embase, Scopus, ScienceDirect, Web of Science, and the Cochrane Library, from inception to December 3, 2025. No restrictions on study design were applied during the initial screening phase in order to maximize search sensitivity. Database-specific search strings were constructed using a combination of Medical Subject Headings (MeSH) terms and free-text keywords related to choroideremia, gene therapy, and adeno-associated viral (AAV) vectors. Core disease-related terms included “choroideremia,” “choroideraemia,” and “*CHM*,” while intervention-related terms comprised “gene therapy,” “genetic therapy,” “AAV,” “adeno-associated virus,” “REP1,” “AAV2-REP1,” and “timrepigene emparvovec.” Search terms are summarized in Supplementary Table S1. To capture clinically relevant outcomes, additional keywords such as “visual acuity,” “microperimetry,” and “fundus autofluorescence” were incorporated where permitted. Boolean operators (AND/OR) were applied to combine concepts appropriately, and search syntax was adapted to the requirements and term limits of each database (including an eight-term limit for Scopus and ScienceDirect). No restrictions were placed on study design during the initial search to maximize sensitivity. Reference lists of included articles and relevant reviews were also manually screened to identify additional eligible studies. Discrepancies in search results or study identification between the two reviewers were resolved through discussion, ensuring a transparent and reproducible study selection process.

### Study selection and screening

Two authors (K. Y. C. and H. C. C.) independently screened the titles and abstracts of all unique records retrieved from the database searches against the predefined eligibility framework based on the PICO (Population, Intervention, Comparator, Outcomes) approach, with any discrepancies resolved through consultation with a third reviewer (C. M. C.). The population of interest included patients with genetically confirmed choroideremia, irrespective of age or disease stage. Studies enrolling mixed retinal dystrophy populations were considered only if choroideremia-specific data could be clearly extracted. The intervention comprised AAV-mediated gene therapy targeting the REP1 gene, including AAV2-REP1 and timrepigene emparvovec administered via subretinal or subfoveal injection. The comparator included untreated contralateral eyes, sham or standard care controls, or baseline measurements in single-arm interventional studies. Eligible outcomes encompassed functional, structural, and safety endpoints, including best-corrected visual acuity (BCVA), retinal sensitivity or microperimetry, fundus autofluorescence (FAF), optical coherence tomography (OCT) parameters, and treatment-related adverse events. Full-text articles were obtained for studies deemed potentially relevant by either reviewer. The same reviewers (K. Y. C. and H. C. C.) then independently assessed full texts for final inclusion. Disagreements at any stage were resolved through discussion and consensus, with arbitration by a third reviewer (C. M. C.) when necessary. Studies were included if they were clinical trials or prospective observational studies evaluating AAV-based gene therapy in choroideremia and reported at least one predefined outcome of interest. Both randomized and non-randomized designs were eligible, reflecting the rarity of the condition and the early-phase nature of many gene therapy trials. Exclusion criteria comprised non-human studies, reviews, editorials, conference abstracts without full data, studies lacking choroideremia-specific outcomes, investigations of non-AAV interventions, or those with insufficient methodological detail. All records retrieved from the database searches were compiled in Zotero software (v. 6.0.36). The software's tools were used to automatically identify and manually merge duplicate records. Where available, additional exploratory structural variables, including cone photoreceptor metrics derived from adaptive optics scanning laser ophthalmoscopy and spectral-domain optical coherence tomography (SD-OCT), were extracted for qualitative synthesis. These exploratory structural assessments were considered alongside cone-mediated functional measures, including microperimetry-based sensitivity testing, when reported.

### Data extraction and quality appraisal

Data extraction was performed using a standardized and piloted data extraction form to ensure consistency and accuracy across included studies. Two reviewers independently extracted data from each eligible article, with cross-verification to minimize errors. Extracted information included study characteristics (author, year, country, study design, sample size, and follow-up duration), participant demographics and disease characteristics, intervention details (AAV vector type, gene construct, dose, and route of administration), and comparator characteristics where applicable. Outcome data encompassed functional measures (BCVA and microperimetry/retinal sensitivity), structural outcomes (FAF and OCT parameters), and safety endpoints (treament-emergent adverse events [TEAEs] and serious adverse events). When multiple follow-up time points were reported within a study, outcome data were extracted for all available follow-up intervals in order to explore potential time-dependent treatment effects of AAV-mediated gene therapy. Each reported follow-up interval (e.g., 3, 6, 12, 24, or ≥48 months) was treated as a separate effect estimate in the quantitative synthesis. Because covariance structures describing correlations between repeated measurements within the same study cohort were not reported in the primary publications, these effect estimates were analyzed as independent observations. This approach allowed the inclusion of longitudinal outcome information across studies while acknowledging the potential statistical dependence between repeated measurements obtained from the same participants. Any discrepancies in extracted data were resolved through discussion and consensus between reviewers, with reference to the original publication to ensure data integrity and completeness. Retinal sensitivity was assessed using fundus-tracked microperimetry, most commonly the MAIA microperimeter. While testing protocols (e.g., grid density, adaptation state) varied between studies, all analyses were based on within-study comparisons between treated and contralateral control eyes conducted under identical testing conditions.

Quality appraisal of included studies followed established tools based on study design. For randomized controlled trials, the Risk of Bias 2.0 (RoB 2)^24^ tool was applied to assess potential bias across domains including randomization, deviations from intended intervention, missing outcome data, outcome measurement, and selective reporting. For non-randomized clinical studies and observational cohorts, the ROBINS-I tool^25^ was used to evaluate bias due to confounding, participant selection, classification of interventions, deviations from intended intervention, missing data, outcome measurement, and reporting bias. Each study was rated as low, moderate, serious, or critical risk of bias according to criteria. Risk-of-bias visualizations were generated using the robvis tool.^26^ The dual-reviewer process and structured appraisal methodology ensured methodological rigor and minimized subjective bias in evaluating study quality. A study-level certainty appraisal was conducted using domains adapted from the Grading of Recommendations Assessment, Development and Evaluation (GRADE) framework. Randomized controlled trials were initially rated as high-certainty evidence, whereas non-randomized studies began as low-certainty evidence. The evidence was downgraded based on the risk of bias, imprecision due to small sample sizes typical of rare disease trials, and methodological limitations of early-phase gene therapy studies.

### Handling missing data

In cases where essential data were missing, the research team attempted to contact the study's corresponding author via email to request clarification or additional information. If a response was not received after two attempts, the analysis proceeded using only the available data. When quantitative data required for meta-analysis were absent, the team calculated estimates from other reported statistics or digitized figures. Any study with significant missing outcome data that could not be resolved was included in the qualitative summary but omitted from the meta-analysis.

### Statistical analysis

All statistical analyses were performed using Comprehensive Meta-Analysis (CMA) software (version 3.7; Biostat, Englewood, NJ, USA). Continuous outcomes, including BCVA, retinal sensitivity measured by microperimetry, preserved RPE area on FAF, and subfoveal choroidal thickness, were pooled using mean differences (MDs) with corresponding 95% confidence intervals (CIs), as outcomes were reported on comparable measurement scales across studies. For dichotomous outcomes and safety endpoints, including treatment-related adverse events and serious adverse events, pooled estimates were calculated using logit-transformed event rates or log odds ratios, depending on data availability, to account for proportional data and stabilize variance. Fixed-effect models were initially considered when statistical heterogeneity was minimal. However, because clinical and methodological heterogeneity across studies was anticipated due to differences in patient populations, baseline disease severity, AAV vector constructs, dosing regimens, and surgical delivery techniques, random-effects models using the DerSimonian–Laird method were ultimately applied for the primary analyses. This approach accounts for both within-study and between-study variability and provides more conservative pooled estimates when synthesizing heterogeneous clinical data. Because the included studies employed heterogeneous comparator structures, including randomized parallel control groups, untreated contralateral fellow eyes used as internal controls, and single-arm designs comparing post-treatment outcomes with baseline measurements, a single uniform comparative estimand could not be defined across all studies. Consequently, pooled effect estimates represent the overall direction and magnitude of change associated with AAV-mediated gene therapy rather than a single standardized comparison between treated and untreated groups. Where available, comparisons between treated and untreated contralateral eyes were prioritized because they provide the most direct within-patient control for disease progression. Randomized parallel-group trials contributed conventional treatment-versus-control effect estimates, whereas single-arm studies contributed estimates reflecting within-eye change from baseline over time. To accommodate these heterogeneous comparator structures, forest plots were presented using neutral effect–size axes without “favors treatment” or “favors control” labels, emphasizing the magnitude and direction of treatment-associated change rather than a uniform causal comparison. When studies reported outcomes at multiple follow-up time points or across different treatment groups, each estimate was included as a separate effect size while retaining the original study identifier. Because several included studies contributed multiple follow-up measurements derived from the same participant cohorts, these effect estimates may not be fully statistically independent. However, covariance structures describing correlations between repeated measurements were not reported in the primary publications, preventing formal adjustment for within-study correlation. Consequently, all available estimates were included as independent observations in the meta-analysis to preserve longitudinal information. Random-effects models (DerSimonian–Laird method) were applied to partially account for variability across estimates and studies. Statistical heterogeneity was assessed using Cochran's Q test and quantified with the I^2^ statistic, with values of 25%, 50%, and 75% representing low, moderate, and high heterogeneity, respectively. Due to formatting limitations of the CMA forest plot outputs, detailed statistical parameters including study weights, Cochran's Q statistic, I^2^ heterogeneity estimates, and associated test statistics could not be fully displayed within the graphical plots; therefore, these values are reported separately in Supplementary Tables S2–S6 for each outcome to ensure complete transparency of the meta-analytic calculations. Subgroup analyses were conducted according to follow-up duration (e.g., 6, 12, 24, and ≥48 months) to explore time-dependent effects. To further investigate potential sources of between-study heterogeneity, random-effects meta-regression analyses were performed using follow-up duration as a moderator variable. The significance of moderators was evaluated using omnibus Q tests, and the proportion of between-study variance explained by the model was assessed using an R^2^ analog. Publication bias was examined through funnel plot inspection, Begg's rank correlation test, Egger's regression test, and the trim-and-fill method where appropriate. A two-sided p value < 0.05 was considered statistically significant for all analyses.

### Role of the funding source

This study received no specific funding. No funder had any role in study design, data collection, data analysis, data interpretation, writing of the report, or the decision to submit the article for publication.

## Results

### Study selection and screening

**Fig. 1:**
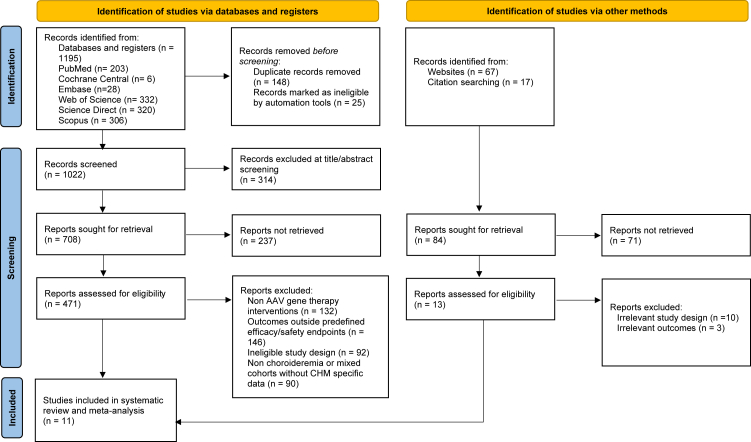
**Preferred Reporting Items for Systematic reviews and Meta-Analyses (PRISMA)2020 flow diagram summarizing identification, screening, eligibility assessment, and final inclusion of studies evaluatingadeno-associated virus (AAV)-mediated gene therapy for choroideremia**.^23^

This PRISMA 2020 flow diagram summarizes the systematic study selection process from both database searching and other methods. In the identification stage via databases and registers, 1195 articles were identified, including 203 from PubMed, 6 from the Cochrane Central Register, 28 from Embase, 332 from Web of Science, 320 from ScienceDirect, and 306 from Scopus. Prior to screening, 148 duplicate records and 25 records marked as ineligible by automation tools were removed, leaving 1022 records for title and abstract screening. During screening, 314 articles were excluded due to lack of relevance, resulting in 708 reports sought for retrieval, of which 237 articles could not be retrieved, leaving 471 reports assessed for eligibility. At the full-text stage, 460 articles were excluded for predefined methodological reasons, including 132 articles with non-AAV gene therapy interventions, 146 articles reporting outcomes outside predefined efficacy or safety endpoints, 92 articles with ineligible study designs, and 90 articles involving non-choroideremia populations or mixed cohorts without extractable CHM-specific data. In parallel, 84 additional records were identified through other methods, including 67 from websites and 17 from citation searching; among these, 71 reports were not retrieved, leaving 13 reports assessed for eligibility, of which all 13 were excluded due to 10 articles with irrelevant study designs and 3 articles with irrelevant outcomes. Ultimately, 11 studies met all eligibility criteria and were included in the final systematic review and meta-analysis (Fig. 1).

### Overview of study characteristics

The systematic review included 11 clinical studies evaluating AAV-mediated gene therapy for choroideremia (CHM), encompassing a total of 308 participants across North America and Europe. The majority of studies were early-phase clinical trials, including Phase I, I/II, and II designs, reflecting the developmental stage of gene therapy in this rare retinal disorder as shown in Table 1. Sample sizes were generally small in early trials (n = 5–15), with larger cohorts observed in later-phase studies, most notably the randomized Phase III trial by MacLaren et al. (2023) involving 169 participants and a Phase II bilateral treatment study by MacLaren et al. (2024) including 66 patients. All studies enrolled adult participants with genetically confirmed choroideremia, consistent with the X-linked inheritance of the disease. Interventions primarily involved single or bilateral subfoveal or subretinal injections of AAV2-based vectors encoding the REP1 gene, with contralateral untreated eyes commonly serving as internal controls. Follow-up durations ranged from 1 month to 5 years, allowing assessment of both short-term safety and longer-term structural and functional outcomes. Key outcomes included BCVA, microperimetry-derived retinal sensitivity, FAF, SD-OCT, and safety endpoints such as TEAEs and immune responses. Overall, the included studies consistently reported that TEAEs were predominantly procedure-associated ocular events, most of which were mild to moderate in severity, alongside largely stable or modestly improved visual and functional outcomes and variable structural changes, highlighting both the therapeutic potential and the heterogeneity of response to AAV-based gene therapy in choroideremia.

**Table 1:** Characteristics of included studies.

Author(s), year	Country/study setting	Study design	Sample size	Intervention details	Comparator/control	Follow-up duration	Outcomes measured	Funding source/sponsor	Key findings pertaining to the treated eyes
Aleman et al., 2022^27^	USA	Prospective, nonrandomized Phase I/II clinical trial	15	Uniocular subfoveal injections of AAV2 vector carrying human CHM-encoding cDNA	Non-injected eyes of the same patients	2 years	Visual acuity, perimetry, SD-OCT, FAF, microperimetry	Spark Therapeutics Inc; additional support from Hope for Vision and Choroideremia Research Foundation	Treated eyes demonstrated overall stable visual acuity and retinal structure with minimal adverse events; however, no significant differences were observed between treated and untreated eyes, and some patients showed possible foveal vulnerability following treatment.
Dimopoulos et al., 2018^28^	Canada	Phase I clinical trial	6	Single subfoveal injection of 0.1 mL rAAV2.REP1	Contralateral untreated eye	2 years	Safety, BCVA change, microperimetry sensitivity, and RPE area by FAF	Canadian Institutes of Health Research, Alberta Innovation Health Services, Canada Foundation for Innovation	One patient experienced a serious adverse event associated with visual decline; however, visual acuity and microperimetry remained stable in most treated eyes, with comparable rates of RPE area loss between treated and untreated eyes. Notably, one untreated fellow eye demonstrated a ≥15 ETDRS-letter gain, indicating that clinically meaningful improvement may also occur in control eyes.
Fischer et al., 2019^29^	Germany	Phase II, open-label randomized clinical trial	6	Single 0.1 mL subretinal injection of AAV2-REP1 (10^11^ genome particles)	Contralateral untreated eye	24 months	BCVA change, microperimetry variables, FAF, SD-OCT	Tistou and Charlotte Kerstan Foundation	Treated eyes maintained or showed modest improvement in visual acuity and retinal sensitivity, with no severe adverse events reported and overall stable structural retinal findings.
Fischer et al., 2020^30^	Germany	Phase II, open-label randomized trial	6	Single 0.1 mL subretinal injection of AAV2-REP1	Contralateral untreated eye	12 months	BCVA, retinal sensitivity, peak retinal sensitivity, gaze fixation area, anatomical endpoints	Tistou and Charlotte Kerstan Foundation; editorial support funded by Nightstar Therapeutics	Treated eyes demonstrated generally stable visual acuity with improvements in retinal sensitivity in several patients, while anatomical retinal changes remained comparable between treated and untreated eyes; adverse events were primarily related to the surgical procedure.
Lam et al., 2018^31^	USA	Phase II clinical trial	6	Single subfoveal injection of AAV2-REP1 (10^11^ genome particles in 0.1 mL)	Contralateral untreated eye	2 years	BCVA change, microperimetry, FAF, SD-OCT, safety	Bascom Palmer Eye Institute, Hope for Vision, National Eye Institute	Most treated eyes maintained or demonstrated modest improvement in visual acuity without serious treatment-related adverse events; however, two patients developed retinal holes in areas of pre-existing retinal thinning.
MacLaren et al., 2014^32^	UK	Clinical trial	6	Subfoveal injection of AAV.REP1	Untreated contralateral eyes	6 months	BCVA, microperimetry, and retinal sensitivity	Health Innovation Challenge Fund (UK Department of Health and Wellcome Trust)	Treated eyes showed improved retinal sensitivity and fixation stability in some patients, particularly those with lower baseline vision, despite temporary retinal detachment associated with the surgical procedure.
MacLaren et al., 2024^33^	UK	Phase II, open-label, prospective, interventional study	66	Bilateral sequential subretinal injections of timrepigene emparvovec, with up to 0.1 mL administered per eye and each dose containing 1 × 10^11^ vector genomes	No control group	12 months	Visual acuity, TEAEs, retinal inflammation, serious surgical complications, and immune responses	Nightstar Therapeutics/Biogen	Bilateral AAV gene therapy was generally well tolerated, with most treated eyes experiencing mild to moderate TEAEs; occasional serious surgical complications were reported but were not associated with sustained vision loss in most cases.
MacLaren et al., 2023^34^	UK	Randomized, masked, Phase III clinical trial	169	Subretinal injection of timrepigene emparvovec (AAV2-REP1 vector), with high-dose (1.0 × 10^11^ vg) and low-dose (1.0 × 10^10^ vg) arms	Untreated control group	12 months	BCVA improvement, adverse events	NightstaRx (acquired by Biogen)	The primary endpoint of visual acuity improvement was not achieved in treated eyes compared with controls, although most adverse events were mild to moderate and related to the surgical procedure.
Morgan et al., 2022^35^	USA	Longitudinal case series	9	Uniocular subfoveal injection of AAV2-hCHM	Contralateral uninjected eyes	1 month	Cone mosaic integrity, OCT imaging, cone-mediated sensitivity	National Institutes of Health, Foundation Fighting Blindness	Treated eyes demonstrated largely preserved cone photoreceptor structure and sensitivity shortly after treatment, although one patient experienced acute foveal cone damage suggesting possible individual susceptibility to surgical or treatment-related effects.
Xue et al., 2018^36^	UK	Non-randomized Phase I/II clinical trial	14	Subretinal injection of AAV2.REP1 vector; low dose	Untreated fellow eyes (internal control)	5 years	BCVA, microperimetry, OCT (retinal thickness), autofluorescence (retinal area), safety (vector shedding, immune response)	Health Innovation Challenge Fund (Wellcome Trust & UK Department of Health)	Treated eyes maintained long-term visual acuity and retinal structure over five years of follow-up, with no systemic immune responses or vector shedding and only rare surgery- or inflammation-related complications.
Zhai et al., 2023^37^	Canada	Extended prospective Phase I/II trial	5	Single subfoveal injection of AAV2-REP1 vector	Untreated fellow eye	5 years	BCVA changes, retinal structure (OCT, FAF), immune response, ASO RNA/protein recovery in fibroblasts	Alberta Innovates; Foundation Fighting Blindness; vector supplied by Nightstar Therapeutics	Some treated eyes experienced retinal complications and visual changes during long-term follow-up, with continued autofluorescence area loss indicating ongoing disease progression despite treatment.

Abbreviations: AAV, adeno-associated virus; AAV2, adeno-associated virus serotype 2; ASO, antisense oligonucleotide; BCVA, best-corrected visual acuity; cDNA, complementary deoxyribonucleic acid; CHM, choroideremia gene; FAF, fundus autofluorescence; hCHM, human choroideremia gene; OCT, optical coherence tomography; rAAV2.REP1, recombinant adeno-associated virus serotype 2 encoding Rab escort protein 1; REP1, Rab escort protein 1; RPE, retinal pigment epithelium; SD-OCT, spectral domain optical coherence tomography; vg, vector genomes, ETDRS, Early Treatment Diabetic Retinopathy Study; TEAE, treatment-emergent adverse event.

### Risk-of-bias assessment

Among the included studies, the majority of early-phase clinical trials were investigator-initiated academic studies supported by governmental or foundation funding, including grants from the National Institutes of Health, Wellcome Trust, and Foundation Fighting Blindness. Later-phase trials evaluating timrepigene emparvovec involved industry sponsorship or collaboration, primarily through Nightstar Therapeutics and subsequently Biogen. Several studies represented mixed sponsorship, where the clinical trial was conducted in academic institutions but investigational vectors or logistical support were provided by industry partners.

**Fig. 2:**
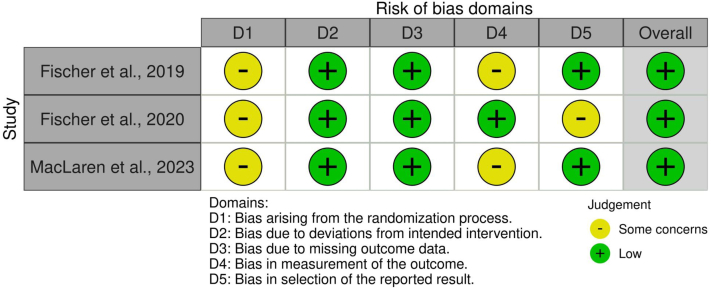
**Risk-of-bias assessment of randomized clinical trials using CochraneRisk of Bias 2 (RoB 2)tool across domains including randomization, deviations, missing data, measurement, reporting**.

The risk of bias in Fig. 2 for the included randomized controlled trials (Fischer et al., 2019; Fischer et al., 2020; and MacLaren et al., 2023) was assessed using the Cochrane RoB 2 tool, evaluating five standard domains. Overall, the randomized trials were judged to present some concerns for risk of bias rather than low risk, primarily due to limited reporting of allocation concealment and the inherent challenges of masking surgical gene therapy interventions. Although outcome measurements such as BCVA and microperimetry are objective and standardized, the absence of full masking and incomplete reporting of certain methodological details resulted in domain-level judgments of “some concerns” in the randomization and outcome measurement domains. According to the RoB 2 framework, the presence of at least one domain rated as “some concerns” results in an overall judgment of some concerns for risk of bias, with some domains raising minor concerns primarily related to trial design constraints inherent to surgical gene therapy studies. Across all RCTs, bias arising from the randomization process (D1) was rated as some concerns, mainly due to limited reporting on allocation concealment and the practical challenges of masking in subretinal surgical interventions. Bias due to deviations from intended interventions (D2) was consistently judged as low risk, as treatments were delivered according to protocol with high adherence and minimal crossover. Missing outcome data (D3) posed a low risk, with complete or near-complete follow-up and transparent reporting of exclusions. For the measurement of outcomes (D4), some concerns were noted in Fischer et al. (2019) and MacLaren et al. (2023), largely because masking of outcome assessors was not always feasible; however, outcomes such as BCVA, OCT, and microperimetry are objective and standardized, mitigating potential measurement bias. Selective reporting (D5) was judged as low risk across trials, with prespecified outcomes consistently reported. Although several trials reported industry collaboration or sponsorship, most studies clearly stated that sponsors had no role in study design, data analysis, or manuscript preparation, and therefore, the risk of bias due to funding source was considered low to moderate.

**Fig. 3:**
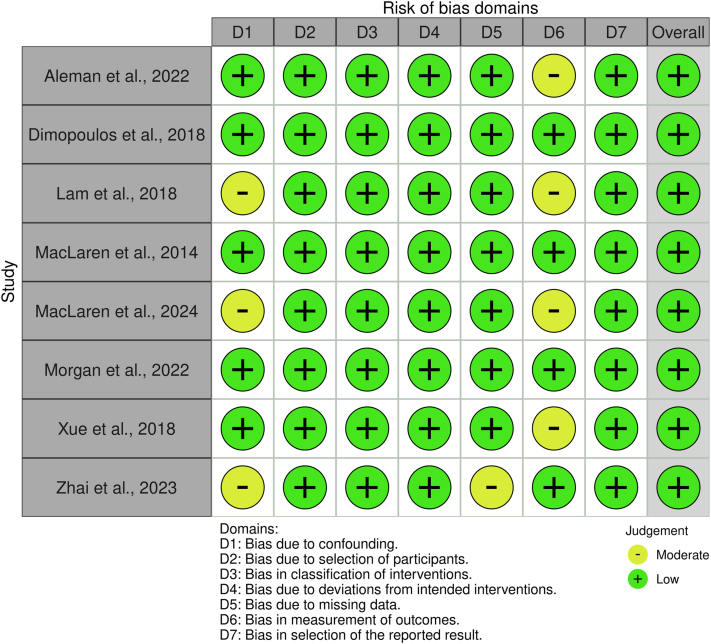
**Risk-of-bias evaluation of non-randomized studies using the Risk Of Bias In Non-randomized Studies of Interventions (ROBINS-I) framework, assessing confounding, participant selection, intervention classification, deviations, missing data, and outcome measurement**.

The risk of bias in Fig. 3 for the included non-randomized and early-phase interventional studies was assessed using the ROBINS-I tool, which evaluates seven domains of bias from confounding through selective reporting. Overall, the majority of studies (Aleman et al., 2022; Dimopoulos et al., 2018; Lam et al., 2018; MacLaren et al., 2014; Morgan et al., 2022; Xue et al., 2018; and Zhai et al., 2023) were judged to be at low overall risk of bias, with moderate concerns in selected domains, primarily related to confounding and outcome measurement. Bias due to confounding (D1) was the most frequent source of concern, particularly in studies using contralateral untreated eyes as internal controls or enrolling patients with heterogeneous disease stages (e.g., Lam et al., 2018; MacLaren et al., 2024; Zhai et al., 2023). While this design reduces inter-individual variability, it cannot fully account for asymmetric disease progression. Selection of participants (D2) and classification of interventions (D3) were consistently judged as low risk, as eligibility criteria were clearly defined and the gene-therapy intervention was well characterized across studies. Bias due to deviations from intended interventions (D4) was also low, reflecting standardized surgical protocols and close postoperative monitoring. Most studies demonstrated low risk of bias from missing data (D5), with high follow-up completeness and transparent reporting of exclusions. Measurement of outcomes (D6) showed moderate concerns in some trials due to the practical inability to mask surgeons and assessors; however, the use of objective endpoints such as BCVA, OCT, FAF, and microperimetry mitigated this risk. Selective reporting (D7) was judged as low across studies, as reported outcomes were generally consistent with study objectives and protocols. While several studies reported support from biotechnology companies developing AAV gene therapies, the majority of trials were investigator-led academic studies. Where industry sponsorship or provision of investigational vectors was reported, this was considered under the bias due to confounding and selective reporting domains, but did not substantially alter the overall risk-of-bias assessment because study methodologies and outcome reporting remained transparent.

The certainty of evidence for the included studies was evaluated using the Grading of Recommendations Assessment, Development and Evaluation (GRADE) framework, incorporating considerations of risk of bias, inconsistency, indirectness, imprecision, and potential publication bias as shown in Table 2. Among the included studies, randomized controlled trials initially contributed higher levels of evidence, whereas non-randomized early-phase clinical trials and observational designs began with lower certainty ratings. The Phase III randomized clinical trial by MacLaren et al. (2023), which included the largest cohort (n = 169), demonstrated moderate to high certainty of evidence, with minimal downgrading primarily related to limitations in masking inherent to surgical interventions. Randomized Phase II trials by Fischer et al. (2019) and Fischer et al. (2020) were downgraded for imprecision due to small sample sizes but retained moderate certainty. In contrast, most early-phase studies, including Aleman et al. (2022), Lam et al. (2018), Xue et al. (2018), and MacLaren et al. (2014), were rated as low or very low certainty because of non-randomized designs and limited participant numbers. Studies with extremely small cohorts, such as Dimopoulos et al. (2018) and Zhai et al. (2023), were further downgraded for very serious imprecision. Overall, these study-level certainty appraisals ranged from very low to moderate‐high. At the body-of-evidence level, the certainty was judged to be moderate for functional outcomes and low to moderate for structural and safety outcomes, reflecting the early clinical development stage of AAV-mediated gene therapy for choroideremia.

**Table 2:** Study-level certainty appraisal using Grading of Recommendations Assessment, Development and Evaluation (GRADE) domains.

Study	Initial level	Risk of bias	Inconsistency	Indirectness	Imprecision	Publication bias	Upgrading factors	Final GRADE certainty
Aleman et al., 2022	Low (observational)	−1 (non-randomized design)	0	0	−1 (small sample n = 15)	0	None	Very low
Dimopoulos et al., 2018	Low	−1 (Phase I, non-randomized)	0	0	−2 (very small sample n = 6)	0	None	Very low
Fischer et al., 2019	High (RCT)	−1 (open-label design)	0	0	−1 (very small sample n = 6)	0	None	Low–moderate
Fischer et al., 2020	High (RCT)	−1 (open-label surgical trial)	0	0	−1 (very small sample n = 6)	0	None	Low–moderate
Lam et al., 2018	Low	−1 (non-randomized)	0	0	−1 (small sample n = 6)	0	None	Very low
MacLaren et al., 2014	Low	−1 (early-phase trial)	0	0	−1 (very small sample n = 6)	0	None	Very low
MacLaren et al., 2024	Low	−1 (open-label interventional design)	0	0	−1 (moderate sample but uncontrolled design)	0	None	Very low–low
MacLaren et al., 2023	High (Phase III RCT)	−1 (surgical masking limitations)	0	0	0 (large sample n = 169)	0	None	Moderate–high
Morgan et al., 2022	Low	−1 (case series design)	0	−1 (very short follow-up 1 month)	−1 (small sample n = 9)	0	None	Very low
Xue et al., 2018	Low	−1 (non-randomized design)	0	0	−1 (moderate sample n = 14)	0	None	Very low–low
Zhai et al., 2023	Low	−1 (non-randomized Phase I/II)	0	0	−2 (very small sample n = 5)	0	None	Very low

Abbreviation: RCT, randomized controlled trial.

### Systematic review results

#### Efficacy outcomes of AAV2-mediated gene therapy

Most studies demonstrated that eyes treated with AAV2-mediated gene therapy maintained visual acuity stability in patients with choroideremia. Thirteen of 15 patients maintained visual acuity within 15 letters of baseline after receiving subfoveal AAV2-h*CHM* injections over a 2-year follow-up period.^27^ Similarly, visual acuity remained stable in all but one patient, and BCVA returned to at least baseline levels by 1 month post-treatment.^38^^,^^39^ However, some studies showed modest improvements. Treated eyes demonstrated small improvements compared to controls, though the overall visual acuity improvements were generally modest.^29^^,^^32^^,^^40^ In other studies, AAV-treated patients experienced notable improvements.^34^^,^^41^

Several papers demonstrated that treated eyes experienced smaller declines in retinal sensitivity compared to controls. There was a smaller decline in mean retinal sensitivity in treated eyes compared to untreated eyes.^32^^,^^36^^,^^41^^,^^42^ However, some studies have shown mixed results, with 4 of 14 patients exhibiting sensitivity losses exceeding baseline variability in the intervention eyes, while 2 patients demonstrated borderline increases in mean sensitivity. Another study reported that intervention eyes showed inter-visit foveal sensitivity differences of −1.5 ± 2.0 dB, while control eyes showed +0.17 ± 1.6 dB at 2 years.^27^

Long-term follow-up studies have revealed sustained benefits, particularly in patients receiving higher doses of gene therapy. Among five patients treated with a high dose of 10^10^ genome particles, treated eyes gained a mean of +4.4 ± 4.2 ETDRS letters at 2 years compared to a mean decline of −2.4 ± 1.9 letters in untreated control eyes, with this effect becoming more pronounced at 3.5 years as treated eyes improved by +8.4 ± 4.7 letters, while control eyes declined by −8.8 ± 3.1 letters.^42^ In another study, structural preservation was demonstrated through cone photoreceptor stability, where cone densities in injected eyes remained stable pre-injection and post-injection.^35^^,^^43^ Similarly, the cone photoreceptor mosaic appeared intact and contiguous in eight of nine treated eyes 1 month post-injection.^44^

However, there was greater central foveal thinning in four of six treated eyes compared with their fellow eyes after 24 months, and a modest decrease in retinal thickness at the central fixation point in treated eyes (−17.1 ± 4.0 μm) compared with untreated eyes (−6.3 ± 2.2 μm).^28^^,^^36^ Structural progression in choroideremia is commonly evaluated using preserved FAF area, which reflects the remaining functional RPE. In some studies, treated eyes showed a mean improvement in BCVA.^45^^,^^46^ In contrast, BCVA in treated eyes showed limited changes for most patients.^30^ At the cellular level, AAV2/2-CBA-REP1 treatment successfully increased prenylation activity and achieved approximately 8-fold higher REP1 protein expression than endogenous levels.^47^

### Safety profile and adverse events

The safety profile of AAV2-mediated gene therapy for choroideremia was characterized by a relatively high frequency of TEAEs, although most events were mild to moderate in severity and consistent with complications commonly associated with subretinal surgical procedures. Across the included trials, ocular pain, vitreous floaters, and transient anterior chamber inflammatory reactions were among the most frequently reported events.^28^ Similarly, other complications included steroid-induced elevations in intraocular pressure in two patients and visually significant posterior capsular cataracts in three patients.^27^ Cataracts occurred more frequently in treated groups overall, affecting 14% of high-dose participants, 12% of low-dose participants, and 5% of controls.^34^^,^^42^

TEAEs occurred more frequently in the treatment groups than in the control group. Safety outcomes revealed that 91% of high-dose recipients, 94% of low-dose recipients, and 51% of control participants experienced TEAEs.^34^ Ocular inflammation-related TEAEs affected 51% of the high-dose group, 47% of the low-dose group, and only 2% of controls in one study, with retinal inflammation-related events affecting 45.5% of participants in another study.^33^^,^^34^ Another study reported 28 adverse events, with 15 being ocular and primarily reflecting expected post-vitrectomy complaints.^29^ In contrast, a different study reported that four participants experienced six serious adverse events in treated eyes.^48^ One study reported a patient who developed acute foveal thinning and another who developed a full-thickness macular hole,^27^ while another study described a case of localized intraretinal immune reaction following treatment.^49^ Serious noninfective retinitis occurred in 3.0% of participants, with two participants developing atrophic retinal holes in nonfunctioning macular regions.^34^ In other studies, patients experienced significant adverse events related to vector administration, and two notable adverse events occurred among six treated subjects in separate studies.^31^^,^^33^^,^^36^^,^^50^ However, AAV2-hCHM was reported to be well tolerated with no adverse events in a few studies.^38^^,^^51^ These findings represent adverse events reported within individual studies included in the systematic review, whereas the overall incidence of treatment-related adverse events was evaluated quantitatively in the subsequent meta-analysis section.

### Influence of baseline disease severity and patient characteristics

Patients with moderate to advanced visual impairment showed that superior therapeutic responses are derived from individual trial-level analyses^29^ and similar early-phase studies. Patients presenting with moderate visual loss at baseline demonstrated larger letter gains in treated eyes with a mean improvement of approximately 5.5 letters.^29^ In contrast, the patient with the best baseline visual acuity demonstrated notable improvement in retinal electrophysiological function in the treated eye.^42^ However, one subject exhibited significant deterioration, including loss of foveal cone sensitivity and a substantial three-line decline in visual acuity.^44^

### Meta-analysis results

#### Retinal sensitivity

**Fig. 4:**
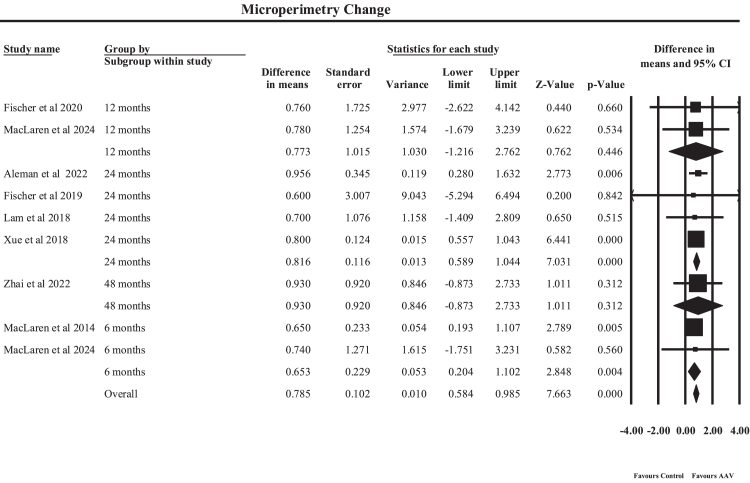
**Forest plot showing pooled random-effects meta-analysis of retinal sensitivity changes measured by microperimetry followingadeno-associated virus (AAV)-mediated gene therapy across follow-up durations**.

A random-effects meta-analysis of human clinical studies as shown in Fig. 4 evaluated the impact of AAV-mediated gene therapy on retinal sensitivity measured by microperimetry, stratified according to follow-up duration. The pooled analysis demonstrated a statistically significant improvement with an overall MD of 0.78 (95% CI: 0.58–0.99, p < 0.001). An MD of 0.78 indicates that, on average, AAV-based gene therapy was associated with an increase of approximately 0.78 dB in retinal sensitivity relative to the comparator used in each individual study. Because the included trials employed heterogeneous comparator designs, including untreated fellow eyes, randomized control groups, and baseline pre-treatment measurements, the pooled estimate reflects the overall magnitude of treatment-associated change rather than a single uniform comparison across studies. Although modest in magnitude, this improvement suggests a measurable enhancement in localized retinal function following gene therapy. Subgroup analyses demonstrated variability across different follow-up durations. At 6 months, the pooled estimate from MacLaren et al. (2014) and MacLaren et al. (2024) showed a significant improvement in retinal sensitivity (MD 0.65, 95% CI: 0.20–1.10, p = 0.004). At 12 months, pooled results from Fischer et al. (2020) and MacLaren et al. (2024) demonstrated a non-significant improvement (MD 0.77, p = 0.45). More pronounced improvements were observed at 24 months, where the pooled estimate from Aleman et al. (2022), Fischer et al. (2019), Lam et al. (2018), and Xue et al. (2018) demonstrated a statistically significant increase in retinal sensitivity (MD 0.82, 95% CI: 0.59–1.04, p < 0.001). Long-term follow-up at 48 months (Zhai et al., 2023) suggested sustained improvement (MD 0.93), although this was not statistically significant due to wider CIs. Overall heterogeneity across studies was minimal, reflecting consistent findings across different trials, while the minor variability observed is likely attributable to differences in subgroup follow-up durations, disease phenotypes, and treatment protocols rather than methodological inconsistency. Clinically, these findings suggest that AAV-mediated gene therapy may improve localized retinal sensitivity over time, particularly with longer follow-up durations. An improvement of approximately 0.78 dB is modest and should be interpreted in light of known test–retest variability in microperimetry for choroideremia; nevertheless, the consistency of the pooled direction of effect supports a small treatment-associated functional signal. These findings support the interpretation that gene therapy may stabilize and modestly enhance retinal sensitivity over time, particularly evident by 24 months. However, the limited magnitude of change also highlights the restricted dynamic range of microperimetry in advanced disease and underscores the need for complementary long-term structural and functional endpoints to fully capture therapeutic benefit.

**Fig. 5:**
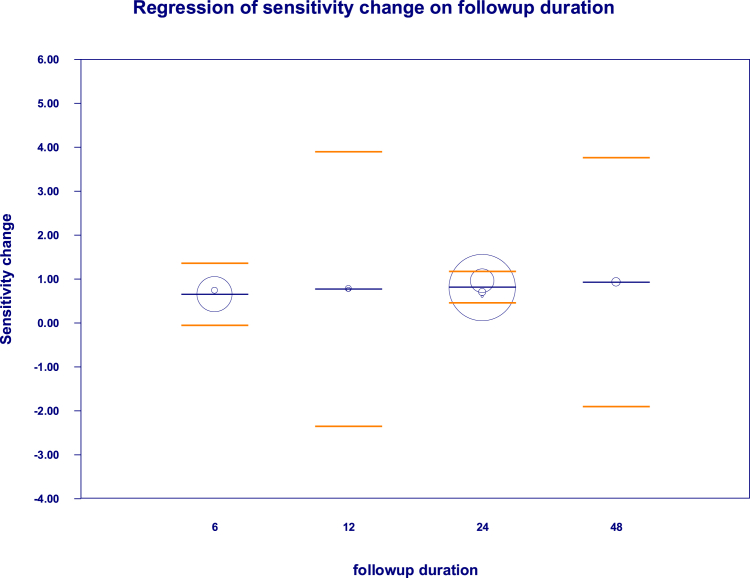
**Meta-regression plot assessing association between follow-up duration and treatment effect on retinal sensitivity afteradeno-associated virus (AAV)-mediated gene therapy in choroideremia**.

A random-effects meta-regression in Fig. 5 was performed to explore whether the duration of follow-up influenced the magnitude of the treatment effect on retinal sensitivity following AAV-mediated gene therapy in choroideremia. The dependent variable was the pooled difference in mean retinal sensitivity between treated and control eyes, while follow-up duration (12, 24, and 48 months) was entered as a categorical moderator, with the intercept representing the reference category. The meta-regression demonstrated a statistically significant intercept (coefficient = 0.65; 95% CI: 0.20–1.10; p = 0.004), indicating an overall positive treatment effect on retinal sensitivity across studies. However, none of the follow-up duration categories showed a statistically significant association with changes in effect size. The coefficients for 12-month (β = 0.12, p = 0.91), 24-month (β = 0.16, p = 0.53), and 48-month follow-up (β = 0.28, p = 0.77) were small and imprecise, with CIs crossing zero. The omnibus test for moderators was non-significant (Q = 0.43, df = 3, p = 0.93), suggesting that follow-up duration did not explain variability in treatment effects. Measures of residual heterogeneity were minimal (τ^2^ = 0.00; I^2^ = 0%), and the model did not explain additional between-study variance compared with the null model (R^2^ analog = 0.00). These findings indicate that the observed improvement in retinal sensitivity is largely consistent across follow-up durations, rather than progressively increasing or diminishing over time within the studied intervals. Clinically, this suggests that AAV-based gene therapy may confer an early and sustained stabilization or modest improvement in retinal sensitivity, with benefits detectable by 12 months and maintained up to 48 months. The absence of a duration-dependent effect likely reflects both limited sample sizes and the relatively slow progression of choroideremia, underscoring the need for longer follow-up and larger trials to clarify long-term functional trajectories.

#### Visual acuity

**Fig. 6:**
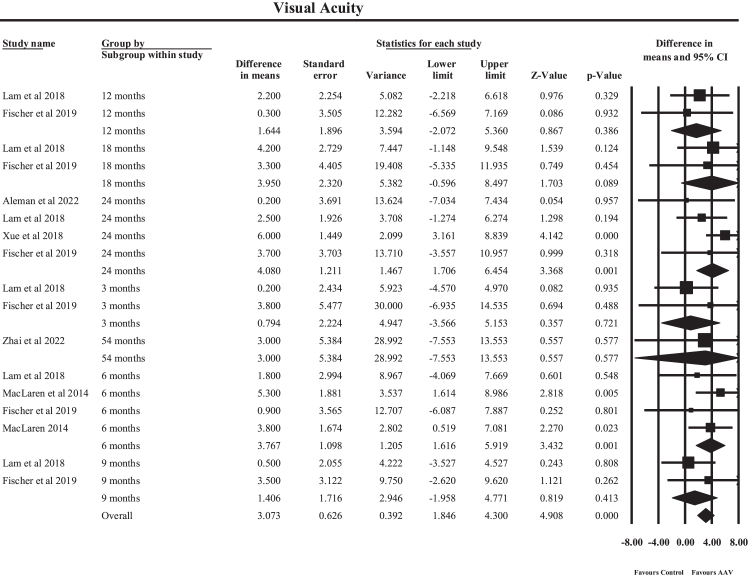
**Forest plot demonstrating pooled random-effects meta-analysis of best-corrected visual acuity changes measured in Early Treatment Diabetic Retinopathy Study (ETDRS) letters following adeno-associated virus (AAV)-mediated gene therapy**.

A random-effects meta-analysis of 17 effect estimates from human clinical studies evaluated the impact of AAV-mediated gene therapy on BCVA, stratified by follow-up duration, as shown in Fig. 6. The pooled analysis demonstrated a statistically significant improvement in BCVA, with an overall MD of 3.07 ETDRS letters (95% CI: 1.85–4.30, p < 0.001). This indicates that patients receiving AAV-based gene therapy experienced an average improvement of approximately three ETDRS letters relative to the comparator applied in each individual study. Because the included studies used heterogeneous comparator frameworks, including untreated contralateral eyes, randomized control groups, and within-eye baseline comparisons, the pooled estimate should be interpreted as the overall magnitude of treatment-associated visual change rather than a single standardized treatment-versus-control comparison, suggesting a modest but measurable functional visual benefit. Subgroup analysis by follow-up duration revealed time-dependent treatment effects. At 3 months, the pooled estimate was not significant (MD 0.79, p = 0.72), indicating minimal early visual change. Statistically significant improvements emerged at 6 months (MD 3.77, 95% CI: 1.62–5.92, p < 0.001), with contributing studies including Lam et al., MacLaren et al., and Fischer et al. Intermediate follow-up intervals such as 9 months (MD 1.41) and 12 months (MD 1.64) showed smaller and non-significant effects, whereas improvement again increased at 18 months (MD 3.95) and 24 months (MD 4.08, 95% CI: 1.71–6.45, p < 0.001), largely driven by studies by Xue et al., Lam et al., Fischer et al., and Aleman et al. Long-term follow-up at 54 months (Zhai et al., 2023) suggested sustained benefit but was based on a single study. Between-study heterogeneity was negligible overall (Q = 10.84, df = 16, p = 0.82; I^2^ = 0%), indicating highly consistent findings across studies. The minimal variability observed is likely attributable to differences in follow-up duration subgroups and study populations rather than methodological inconsistency. Clinically, the pooled improvement of approximately three ETDRS letters suggests visual stabilization rather than dramatic functional recovery, which is nevertheless meaningful in a progressive degenerative disorder such as choroideremia. The progressive increase in effect size from early to mid-term follow-up supports the hypothesis that AAV gene therapy may initially stabilize retinal degeneration and subsequently confer incremental functional gains over time.

#### Preserved retinal pigment epithelium area on fundus autofluorescence

**Fig. 7:**
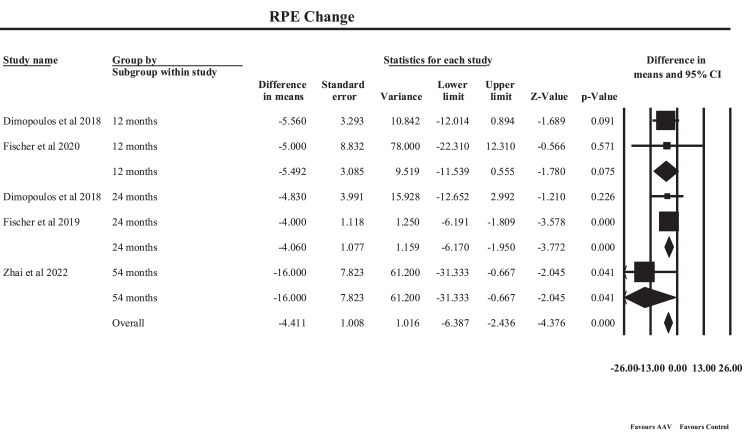
**Forest plot illustrating pooled treatment effects on preserved retinal pigment epithelium(RPE)area measured by fundus autofluorescence(FAF)afteradeno-associated virus (AAV)-mediated gene therapy**.

A random-effects meta-analysis of five human study estimates assessed the effect of AAV-mediated gene therapy on the preserved RPE area measured using FAF, stratified by follow-up duration as shown in Fig. 7. The pooled analysis demonstrated a statistically significant overall effect with an MD of −4.41 (95% CI: −6.39 to −2.44, p < 0.001). An MD of −4.41 indicates that, on average, the decline in preserved RPE area was 4.41 units lower in treated eyes compared with expected progression, suggesting that AAV-mediated gene therapy slows the rate of RPE degeneration. Because FAF-derived RPE area loss reflects disease progression in inherited retinal disorders, a smaller negative change represents greater preservation of retinal structure over time. Subgroup analyses based on follow-up duration revealed variable effects. At 12 months, the pooled estimate from Dimopoulos et al. (2018) and Fischer et al. (2020) showed a non-significant reduction in RPE loss (MD −5.49, p = 0.075). At 24 months, the pooled analysis of Dimopoulos et al. (2018) and Fischer et al. (2019) demonstrated a significant protective effect (MD −4.06, 95% CI: −6.17 to −1.95, p < 0.001). Long-term follow-up at 54 months (Zhai et al., 2023) also showed a significant reduction in RPE degeneration (MD −16.0, p = 0.041), suggesting sustained structural preservation with prolonged follow-up. Between-study heterogeneity was negligible (Q = 2.47, df = 4, p = 0.65; I^2^ = 0%), indicating consistent findings across studies. Any minor variability observed is likely attributable to differences in subgroup follow-up durations, study populations, and disease phenotypes rather than methodological inconsistency. Clinically, these findings suggest that AAV-mediated gene therapy may slow the structural progression of retinal degeneration by preserving RPE integrity, a key determinant of photoreceptor survival. This structural preservation provides important mechanistic support for the functional benefits observed in visual acuity and retinal sensitivity outcomes, highlighting the potential of gene therapy as a disease-modifying treatment in inherited retinal disorders. Although short-term FAF changes are subtle, the progressive signal observed at 24 months and beyond suggests that AAV gene therapy may slow structural disease progression, even when functional gains are modest. These findings support FAF as a valuable long-term structural endpoint, particularly for capturing therapeutic benefit beyond early postoperative periods.

#### Treatment-emergent adverse events

**Fig. 8:**
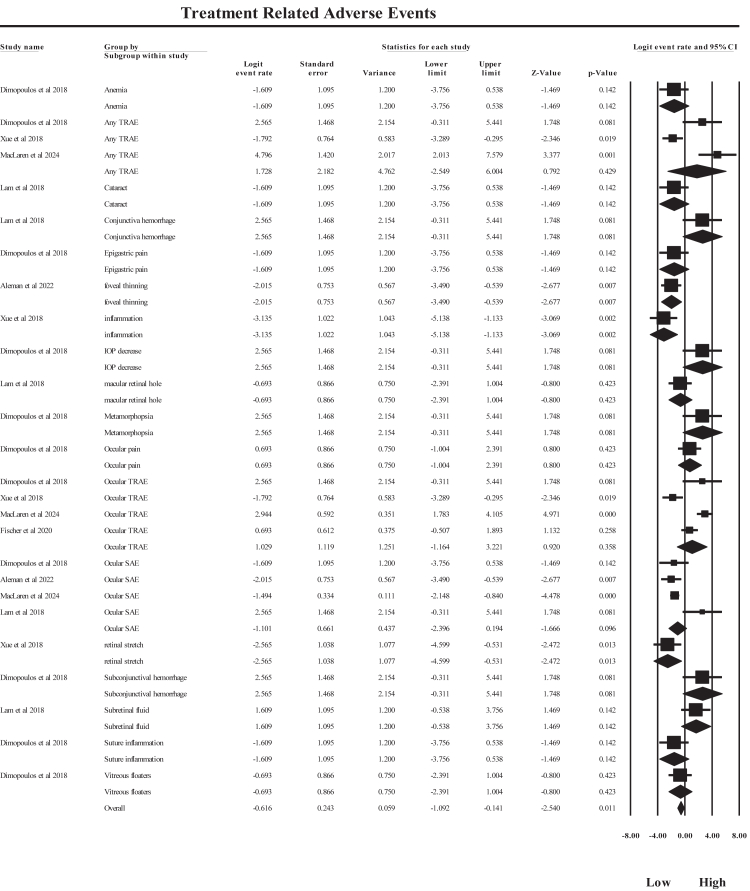
**Forest plot summarizing pooled treatment-emergent adverse event (TEAE) frequencies across clinical trials evaluating adeno-associated virus (AAV)-mediated gene therapy for choroideremia**.

A meta-analysis of 26 TEAE outcomes across human studies evaluating AAV-mediated retinal gene therapy demonstrated a statistically significant pooled estimate under a random-effects model (logit effect size = −0.616, 95% CI: −1.092 to −0.141, p = 0.011), as shown in Fig. 8. As several included studies reported TEAEs only in treated cohorts without comparable control groups, the pooled analysis was conducted using logit-transformed event rates rather than relative risk estimates. This approach allows the synthesis of adverse-event frequencies across studies with heterogeneous reporting formats while preserving comparability of effect estimates. The pooled logit estimate of −0.616 corresponds to an approximate event probability of about 35%, indicating that TEAEs occurred in roughly one-third of treated participants across studies. Adverse-event severity classifications were based on the definitions reported in the original trials corresponding to Common Terminology Criteria for Adverse Events (CTCAE) clinical trial grading systems, where mild events do not interfere with daily activities, moderate events require medical treatment, and severe events involve significant ocular complications or require surgical intervention. Detailed reporting of individual TEAEs from the included studies is provided in Supplementary Table S7. The pooled logit estimate represents the overall frequency of reported adverse events among treated participants, reflecting the safety profile observed across clinical trials rather than a direct comparison demonstrating protection relative to untreated control eyes. Across studies, most reported adverse events were procedure-related, including subconjunctival hemorrhage, transient inflammation, or mild ocular discomfort. Serious ocular adverse events (SAEs) showed a pooled logit estimate of −1.42, with contributing evidence from MacLaren et al., 2024, Aleman et al., 2022, and Dimopoulos et al., 2018. The pooled logit estimate for SAEs (−1.42) corresponds to an approximate probability below 20%, indicating that severe complications were relatively uncommon compared with overall treatment-emergent events. Specific outcomes such as retinal stretch (logit −2.56, p = 0.013), foveal thinning (logit −2.01, p = 0.007), and ocular inflammation (logit −3.14, p = 0.002) also demonstrated statistically significant pooled estimates. In contrast, broader categories such as any TRAE and ocular TRAE showed non-significant pooled effects, reflecting variability across studies including Xue et al., 2018, Dimopoulos et al., 2018, MacLaren et al., 2024, and Fischer et al., 2020. Individual studies demonstrated variable event frequencies. MacLaren et al., 2024 reported a high incidence of TEAEs, reflected by a positive logit estimate (logit 4.79), indicating higher event rates within treated cohorts due to surgical and inflammatory events associated with subretinal delivery. Substantial heterogeneity was observed overall (I^2^ = 80.2%, Q = 126.18, p < 0.001), which is expected given the inclusion of multiple adverse-event categories and differences in vector constructs, delivery routes, disease indications, and follow-up durations. This heterogeneity is therefore primarily attributable to differences between adverse-event subgroups rather than methodological inconsistency. Clinically, these findings indicate that TEAEs are relatively common following AAV-mediated gene therapy, largely reflecting the surgical nature of subretinal vector delivery. However, the majority of events were mild or moderate in severity and were manageable with standard postoperative care. Severe complications such as retinal holes, inflammatory retinitis, or structural macular changes were reported in a minority of treated eyes and occurred substantially less frequently than mild procedure-related events. These observations suggest that the safety profile of AAV-mediated retinal gene therapy is best characterized as procedure-associated and manageable within specialized surgical centers rather than inherently risk-free.

#### Subfoveal choroidal thickness

**Fig. 9:**
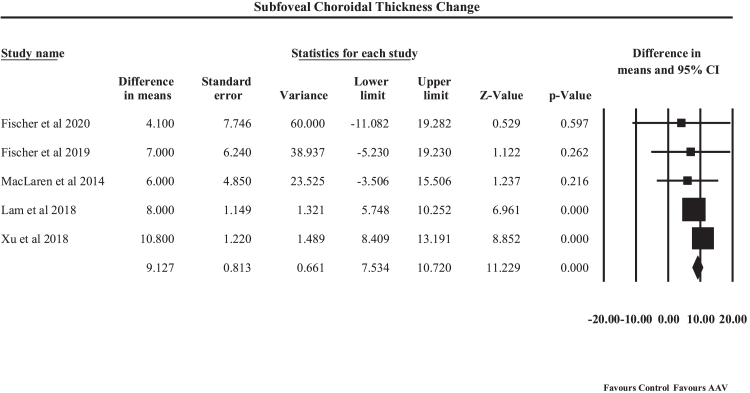
**Forest plot showing pooled changes in subfoveal choroidal thickness measured by optical coherence tomography(OCT)followingadeno-associated virus (AAV)-mediated gene therapy in choroideremia**.

A random-effects meta-analysis of five human studies evaluated the effect of AAV-mediated gene therapy on subfoveal choroidal thickness, an anatomical parameter associated with retinal perfusion and structural integrity, as shown in Fig. 9. The pooled analysis demonstrated a statistically significant increase in subfoveal choroidal thickness with an MD of 9.13 μm (95% CI: 7.53–10.72, p < 0.001). An MD of 9.13 μm indicates that, on average, AAV-based gene therapy was associated with an increase of approximately 9 μm in subfoveal choroidal thickness relative to the comparator used within each contributing study. Because some studies compared treated eyes with untreated fellow eyes, while others assessed within-eye changes from baseline, the pooled estimate represents the overall magnitude and direction of treatment-associated structural change rather than a single uniform treatment-versus-control contrast, suggesting improved choroidal structural stability following treatment. Individual contributing studies included Fischer et al. (2020, 2019), MacLaren et al. (2014), Lam et al. (2018), and Xue et al. (2018). Among these, Lam et al. (2018) and Xue et al. (2018) demonstrated the largest and statistically significant improvements, with mean increases of 8.0 μm and 10.8 μm, respectively. In contrast, Fischer et al. (2020, 2019) and MacLaren et al. (2014) reported smaller and non-significant changes, although the direction of effect consistently favored increased choroidal thickness following therapy. Between-study heterogeneity was negligible (Q = 3.79, df = 4, p = 0.43; I^2^ = 0%), indicating high consistency of findings across the included studies. The small variability observed is likely attributable to differences in study subgroups, follow-up durations, underlying disease phenotypes, and treatment protocols rather than methodological inconsistency. Clinically, increased subfoveal choroidal thickness may reflect improved choroidal perfusion and stabilization of the outer retinal environment, which is essential for photoreceptor survival and RPE function. These structural changes complement the functional improvements observed in visual acuity and microperimetry outcomes, suggesting that AAV-mediated gene therapy may contribute to both anatomical preservation and functional stabilization in inherited retinal diseases, supporting its role as a potential disease-modifying therapeutic strategy.

#### Publication bias

Publication bias assessment (Supplementary Figures S1–S5) showed that functional and structural efficacy outcomes appear robust, while caution is warranted when interpreting safety outcomes, where heterogeneity due to the subtype variability contributes to asymmetry rather than true publication bias. For retinal sensitivity (9 studies), neither Begg's test (Kendall's τ = −0.25, p = 0.35) nor Egger's test (intercept ≈ 0, p = 0.99) indicated funnel plot asymmetry, and the Trim-and-Fill method suggested no missing studies. A large fail-safe N (54) further supports robustness against unpublished null results. Similarly, BCVA (17 studies) showed no significant publication bias on Begg's test (τ = −0.14, p = 0.43). Although Egger's test was borderline on the one-tailed analysis, it was non-significant on the two-tailed test, and the Trim-and-Fill method did not impute missing studies, indicating stable pooled estimates. In contrast, preserved RPE area (5 studies) demonstrated statistically significant asymmetry on both Begg's test (τ = −0.90, p = 0.027) and Egger's test (p < 0.001), suggesting potential publication bias. However, the Trim-and-Fill analysis did not identify missing studies, and pooled effect estimates remained unchanged, indicating a limited impact on the overall conclusion. For TRAE (26 studies), Begg's test indicated significant asymmetry (τ = 0.47, p = 0.0025), and Egger's test showed borderline evidence of bias. The Trim-and-Fill method suggested several potentially missing studies, particularly influencing random-effects estimates, reflecting heterogeneity in adverse event reporting rather than selective non-publication alone. Finally, subfoveal choroidal thickness (5 studies) showed no evidence of publication bias on Begg's test (τ = 0, p = 1.00), with only borderline Egger's results and no missing studies identified by the Trim-and-Fill method.

## Discussion

This comprehensive synthesis of functional, structural, and safety outcomes provides convergent evidence that AAV-mediated gene therapy for choroideremia is associated with modest disease-modifying effects, with a generally manageable, procedure-associated safety profile. Across nine studies, treated eyes demonstrated a consistent improvement in retinal sensitivity of approximately 0.78 dB. Although this effect is modest and must be interpreted in light of known test–retest variability in microperimetry for choroideremia, the consistent direction of pooled effect supports a small treatment-associated functional signal. Importantly, both subgroup analyses and meta-regression indicated that this benefit is early and sustained, rather than strongly time-dependent, suggesting stabilization of retinal function rather than progressive short-term gain. This aligns well with the natural history of choroideremia, a slowly progressive disorder in which functional preservation is itself clinically valuable. Visual acuity outcomes further reinforced this interpretation, with an overall gain of ∼3 ETDRS letters, particularly pronounced at mid-term follow-up (24 months). While modest in magnitude, such gains are meaningful in a condition where untreated eyes typically exhibit gradual decline. Structural analyses provided complementary evidence of disease modification, as treated eyes showed slower loss of preserved RPE area on FAF and an approximately 9 μm relative increase or preservation of subfoveal choroidal thickness, suggesting attenuation of underlying chorioretinal degeneration. Together, these findings support the concept that AAV gene therapy may exert its primary benefit through slowing structural progression, with secondary stabilization of visual function. Safety analyses indicated that TEAEs occurred relatively frequently across clinical trials, particularly in studies involving subretinal surgical delivery of the gene therapy vector. However, the majority of reported events were mild or moderate in severity and were primarily related to the surgical procedure rather than vector-specific toxicity. Serious ocular complications, including retinal holes, inflammatory reactions, or macular structural changes, were reported but occurred in a relatively small proportion of treated eyes. These findings suggest that the safety profile of AAV-mediated gene therapy is largely driven by procedural risk rather than biological toxicity of the vector itself.

Although heterogeneity was high for adverse event outcomes, this largely reflected the aggregation of biologically distinct event types rather than conflicting safety signals.

In the context of a rare, progressive blinding disorder with no established disease-modifying therapies, a TEAE rate of approximately 35%, predominantly composed of mild to moderate, procedure-related events, and an SAE probability below 20% can be considered clinically acceptable, particularly when managed within experienced surgical centers. Publication bias assessments were generally reassuring for retinal sensitivity, BCVA, and subfoveal choroidal thickness, whereas asymmetry for preserved RPE area and adverse-event outcomes warrants cautious interpretation. Clinically, these results suggest that AAV gene therapy is best viewed not as a restorative intervention, but as a disease-modifying strategy capable of preserving retinal structure and function, particularly when administered before advanced degeneration. The findings underscore the importance of early patient selection, long-term follow-up, and the use of complementary functional and structural endpoints to fully capture therapeutic benefit in future trials and real-world implementation.

Interpretation of treatment effects must also be considered within the context of the natural history of choroideremia. Longitudinal observational studies, including the NIGHT natural history study, have shown that BCVA may remain relatively stable over short- to mid-term follow-up despite ongoing structural retinal degeneration. This apparent dissociation between functional and structural disease progression suggests that BCVA may represent a relatively insensitive endpoint for detecting early therapeutic effects in choroideremia. Structural biomarkers such as preserved FAF area, ellipsoid zone integrity on OCT, and localized retinal sensitivity assessed by microperimetry may therefore provide more sensitive indicators of disease modification. In this context, the modest improvements in BCVA observed in the present meta-analysis are most appropriately interpreted as stabilization of visual function rather than dramatic restoration of vision.

Minor fluctuations in visual acuity or retinal sensitivity were generally observed in untreated fellow eyes in several studies using contralateral-eye controls, and many of these changes were interpreted as test–retest variability, learning effects during repeated microperimetry assessments, or other sources of inter-eye variability rather than a therapeutic effect of gene therapy. However, this pattern was not universal. Dimopoulos et al. reported a ≥15 ETDRS-letter gain in one untreated eye, indicating that clinically meaningful improvement may occasionally occur in untreated fellow eyes. This finding highlights an important limitation of contralateral-eye control designs, because inter-eye variability may confound interpretation of treatment effects.

Although microperimetry protocols varied across studies with respect to adaptation state and grid configuration, all analyses were based on within-study comparisons between treated and contralateral control eyes assessed under identical conditions. Consequently, the pooled MDs reflect relative treatment effects rather than absolute sensitivity values and are unlikely to be materially biased by inter-study protocol variability.

The preclinical studies by Tolmachova et al. (2013)^47^ and Vasireddy et al. (2013)^52^ provide strong biological support for the clinical effects observed in this meta-analysis. Tolmachova et al.^47^ demonstrated that subretinal delivery of an AAV2/2-CBA-REP1 vector achieved robust REP1 expression in photoreceptors and RPE, restoring Rab prenylation and improving retinal function in patient-derived fibroblasts and human retinal explants. Vasireddy et al.^52^ similarly showed efficient vector penetration with widespread REP1 expression in adult human retinal explants without structural toxicity. These mechanistic findings align closely with the clinical evidence of stabilized or modestly improved retinal sensitivity along with slower RPE loss and preserved subfoveal choroidal thickness in treated eyes. The absence of retinal toxicity in preclinical models parallels the clinical observation that most reported adverse events were procedure-associated and mild to moderate in severity, whereas SAEs were less frequent. Collectively, these translational data suggest that the modest clinical benefits observed reflect disease stabilization rather than insufficient vector efficacy, underscoring the importance of earlier intervention to maximize therapeutic impact in choroideremia.

The study reveals a nuanced therapeutic profile for AAV2-mediated gene therapy in choroideremia, characterized by modest efficacy and procedure-associated safety considerations. Visual acuity outcomes provide insight into the therapy's mechanism and clinical utility. In our meta-analysis, the largest and most precise effect estimates were observed at 24 months, indicating a temporal pattern of functional stabilization or modest improvement. A delayed therapeutic response has been proposed in prior translational studies, potentially reflecting gradual AAV-mediated transgene expression and REP1 protein accumulation^53^; however, this mechanistic explanation remains inferential and is not directly demonstrated by the present clinical data.

The modest but consistent improvements in retinal sensitivity, together with small gains in BCVA, likely reflect both the limited dynamic range and test–retest variability of microperimetry in advanced choroideremia rather than a true dissociation between functional and visual outcomes. This may reflect limitations in measurement sensitivity or suggest that functional improvements primarily manifest through central visual pathways rather than generalized enhancement of retinal sensitivity. On the other hand, preservation of cone photoreceptor structure in individual studies supports cellular-level therapeutic activity.

The relatively low incidence of SAEs, despite frequent mild to moderate TEAEs, supports a generally manageable safety profile in specialized surgical settings. The occurrence of TEAEs largely reflects the procedural risks associated with subretinal vector delivery rather than intrinsic vector toxicity. On the other hand, the predominance of surgery-related complications, including retinal detachment, macular holes, and inflammatory responses, indicates therapy-specific risks. While our meta-analysis demonstrates modest and consistent functional and structural benefits of AAV-mediated gene therapy, it does not directly evaluate differences in delivery techniques or patient–delivery system matching. The importance of optimizing delivery methods and tailoring intervention strategies to patient characteristics has been emphasized in prior mechanistic and surgical reviews,^54^^,^^55^ and our findings are broadly consistent with these observations.

Patient heterogeneity in baseline disease severity may influence therapeutic response. Several individual studies qualitatively suggested that eyes with greater residual retinal structure and function at baseline appeared more likely to demonstrate functional stabilization or modest improvement; however, this meta-analysis did not perform formal stratification by baseline disease severity, and such observations should be considered hypothesis-generating rather than confirmatory. This suggests an optimal therapeutic window in which sufficient retinal architecture remains to permit functional stabilization, while measurable progression risk remains sufficient to detect treatment effects over follow-up.

This study comprehensively evaluates AAV2-mediated gene therapy for choroideremia, including both short- and long-term follow-up data, and provides valuable insights into time-dependent patterns in treatment effects across different follow-up durations. Additionally, the safety analysis encompasses a relatively large cumulative patient sample for a rare disease; however, variations in surgical technique, perioperative management, vector dosing, and reporting standards across studies limit the extent to which these findings can be considered broadly generalizable. Instead, the results indicate a consistent safety profile across diverse clinical and surgical contexts, rather than uniform applicability.

Although both randomized and non-randomized studies were included, the limited number of randomized controlled trials and the predominance of early-phase interventional studies restricted the feasibility of conducting a statistically meaningful subgroup meta-analysis based on study design. Risk-of-bias assessments using RoB 2 for randomized trials and ROBINS-I for non-randomized studies were therefore performed to account for methodological differences across study types. Some included studies contributed multiple effect estimates corresponding to different follow-up durations or treatment subgroups derived from the same underlying patient cohorts. Because covariance matrices describing correlations between repeated measurements were not reported in the primary studies, these estimates could not be statistically adjusted for within-study dependence. Consequently, effect sizes were analyzed as independent observations within the meta-analysis. Although this approach allows incorporation of longitudinal outcome data, it may slightly underestimate uncertainty around pooled estimates and should therefore be interpreted with caution.

However, moderate heterogeneity existed across studies with respect to vector design and delivery strategies, including differences in AAV2-based constructs (e.g., REP1 expression driven predominantly by CBA promoters), administered vector doses, and unilateral versus bilateral subretinal injection approaches. Although these factors were not amenable to formal subgroup or meta-regression analyses due to the small number of eligible trials and overlapping cohorts, they represent biologically relevant sources of variability that may influence treatment response and therefore warrant consideration when interpreting pooled estimates. The predominance of small Phase I/II trials limits statistical power, while the reliance on internal controls (contralateral eyes) may underestimate natural disease progression. The included studies differed in follow-up duration, ranging from short-term assessments at 6–12 months to long-term follow-up extending up to 5 years, and employed heterogeneous outcome measurement methodologies, including varying BCVA testing protocols, microperimetry platforms and testing conditions, and differences in FAF quantification. This methodological variability introduces analytical complexity and limits direct comparability across trials. Additionally, variability in patient selection criteria and surgical approaches across the included studies, as reported in trial methodologies, which may contribute to differences in observed outcomes but were not formally evaluated in this analysis.

The findings of this meta-analysis suggest that AAV-mediated gene therapy in choroideremia is associated with modest but consistent functional and structural signals, including stabilization of retinal sensitivity, small improvements in BCVA, and reduced progression of RPE loss, alongside a clinically acceptable safety profile dominated by transient, procedure-related ocular adverse events. Clinically, these results support the interpretation that gene therapy may act primarily as a disease-modifying intervention rather than a restorative treatment, particularly in patients with residual foveal structure. The observed effects are unlikely to translate into dramatic short-term visual gains but may be meaningful in the context of a slowly progressive retinal dystrophy where untreated eyes typically decline over time. Importantly, the consistency of effects across studies with minimal heterogeneity reinforces the biological plausibility of treatment benefit, while the limited magnitude of change underscores the need for careful patient selection and realistic counseling. Overall, these findings inform clinicians about expected outcomes and safety considerations, aiding shared decision-making while highlighting the exploratory nature of current evidence.

Future research in choroideremia gene therapy should prioritize adequately powered, randomized, and masked clinical trials with standardized functional and structural endpoints to clarify the true magnitude and durability of treatment effects. Harmonization of outcome measures, particularly microperimetry protocols, visual acuity testing, and FAF quantification, is essential to enable meaningful cross-study comparisons and robust meta-analytical synthesis. Longer follow-up periods extending beyond 2–3 years are needed to determine whether early functional stabilization translates into sustained disease modification in this slowly progressive condition. Additionally, future studies should incorporate stratified analyses based on baseline disease stage and foveal integrity to identify patient subgroups most likely to benefit from intervention. Integration of advanced imaging biomarkers and patient-reported outcome measures may further improve sensitivity to change. Collectively, these approaches will help move the field from exploratory efficacy signals toward a clearer understanding of clinical relevance and optimal trial design.

In conclusion, this study provides the most comprehensive quantitative synthesis to date of the efficacy and safety of AAV2-mediated gene therapy for choroideremia, directly addressing the study aim of evaluating whether this intervention confers meaningful functional and structural benefits while maintaining a safety profile that can be considered clinically acceptable in the context of a progressive blinding disorder with no established disease-modifying therapies. By integrating data from 11 clinical studies encompassing early-phase trials through a randomized phase III trial, our findings demonstrate that AAV gene therapy exerts a consistent disease-modifying effect rather than dramatic visual restoration. Functionally, treated eyes showed a statistically significant improvement in retinal sensitivity (+0.78 dB) and a modest improvement or stabilization in BCVA of approximately 3 ETDRS letters relative to the comparator or baseline structure used in the contributing studies. These effects were characterized by early stabilization followed by modest improvement, particularly evident by 24 months, aligning with the slow natural progression of choroideremia. Meta-regression confirmed that treatment benefits were sustained across follow-up durations, supporting the interpretation that AAV therapy preserves retinal function rather than inducing time-dependent escalation of effect. Structurally, gene therapy was associated with reduced loss of preserved RPE area on FAF and an approximately 9 μm relative preservation of subfoveal choroidal thickness, indicating attenuation of underlying chorioretinal degeneration. These structural findings provide biological support for the observed functional stabilization and support the role of FAF and choroidal metrics as sensitive long-term endpoints. Safety analyses indicated that TEAEs were common across clinical trials, reflecting the surgical nature of subretinal vector administration. Nevertheless, most reported events were mild or moderate in severity and were manageable with standard postoperative care. Serious ocular complications were relatively infrequent and were generally associated with surgical delivery rather than intrinsic vector toxicity. Although heterogeneity was high for adverse-event outcomes, this reflected variability in event subtype reporting rather than contradictory safety signals. Publication bias assessments further supported the robustness of functional and structural efficacy findings. Collectively, these results indicate that AAV2-mediated gene therapy should be regarded as a disease-modifying intervention that slows structural degeneration and stabilizes visual function, particularly when administered before advanced foveal loss. Future trials should emphasize earlier intervention, standardized outcome measures, optimized delivery techniques, and long-term follow-up to fully realize and accurately quantify therapeutic benefit in choroideremia.

## Contributors

K.-Y. C. contributed to conceptualization, methodology, software, investigation, validation, writing the original draft, visualization, and formal analysis, and accessed and verified the underlying data. H.-C. C. contributed to conceptualization and methodology, and accessed and verified the underlying data. C.-M. C. contributed to methodology, investigation, validation, supervision, and project administration. All authors reviewed and approved the final manuscript.

## Data sharing statement

All data generated or analyzed during this study are included in this published article and its tables and figures. No additional datasets are available.

## Declaration of interests

The authors declare that they have no competing interests.

## Appendix A Supplementary data

Supplementary Tables

Translated Abstract
