# Supplementary material for: Efficacy and safety of AAV-mediated gene therapy for choroideremia: a systematic review and meta-analysis
Source: eClinicalMedicine. 2026 May 11;95:103923. doi: 10.1016/j.eclinm.2026.103923 (PMC13191274; doi:10.1016/j.eclinm.2026.103923)
Supplement: Supplementary Figures [file mmc1.docx]

**Supplementary Figure**

Supplementary Figure 1. Funnel plot assessing publication bias for retinal sensitivity outcomes.

Supplementary Figure 2. Funnel plot assessing publication bias for best corrected visual acuity outcomes.

Supplementary Figure 3. Funnel plot assessing publication bias for preserved retinal pigment epithelium area outcomes.

Supplementary Figure 4. Funnel plot assessing publication bias for treatment related adverse event outcomes. Supplementary Figure 5. Funnel plot assessing publication bias for subfoveal choroidal thickness outcomes.
