# Supplementary material for: Efficacy and safety of AAV-mediated gene therapy for choroideremia: a systematic review and meta-analysis
Source: eClinicalMedicine. 2026 May 11;95:103923. doi: 10.1016/j.eclinm.2026.103923 (PMC13191274; doi:10.1016/j.eclinm.2026.103923)
Supplement: Supplementary Tables [file mmc2.docx]

Supplementary Tables:

Supplementary Table 1. Search strategy keywords and Boolean logic used across databases.

| Database | Keywords used |
| --- | --- |
| PubMed | ("Choroideremia"[MeSH Terms] OR ("Choroideremia"[MeSH Terms] OR "Choroideremia"[All Fields] OR "choroideraemia"[All Fields]) OR ("chm"[Journal] OR "chm"[All Fields])) AND ("genetic therapy"[MeSH Terms] OR ("genetic"[All Fields] AND "therapy"[All Fields]) OR "genetic therapy"[All Fields] OR ("gene"[All Fields] AND "therapy"[All Fields]) OR "gene therapy"[All Fields] OR "AAV"[All Fields] OR "adeno-associated virus"[All Fields]) AND ("REP1"[All Fields] OR ("chm"[Journal] OR "chm"[All Fields]) OR "AAV2-REP1"[All Fields] OR "timrepigene emparvovec"[All Fields]) |
| Scopus | TITLE-ABS-KEY (choroideremia) AND TITLE-ABS-KEY ("gene therapy") AND TITLE-ABS-KEY (AAV) AND TITLE-ABS-KEY (REP1) AND TITLE-ABS-KEY ("visual acuity") AND TITLE-ABS-KEY (microperimetry) AND TITLE-ABS-KEY ("fundus autofluorescence") |
| Science Direct | "choroideremia" AND ("gene therapy"OR "AAV" OR "REP1") AND ("visual acuity"OR microperimetry OR "fundus autofluorescence") |
| Web of Science | (choroideremia OR CHM) AND ("gene therapy" OR AAV OR "adeno-associated virus" OR REP1 OR "AAV2-REP1" OR timrepigene) |
| Cochrane Library | (choroideremia OR CHM) AND (gene therapy OR AAV OR "adeno-associated virus") AND (REP1 OR "AAV2-REP1" OR timrepigene) AND (visual acuity OR microperimetry OR "fundus autofluorescence") |
| EMBASE | ('choroideremia'/exp OR choroideremia:ti,ab OR choroideraemia:ti,ab OR CHM:ti,ab) AND ('gene therapy'/exp OR 'genetic therapy':ti,ab OR 'gene therapy':ti,ab OR AAV:ti,ab OR 'adeno associated virus':ti,ab) AND (REP1:ti,ab OR 'AAV2-REP1':ti,ab OR 'timrepigene emparvovec':ti,ab) AND ('visual acuity'/exp OR 'visual acuity':ti,ab OR microperimetry:ti,ab OR 'fundus autofluorescence':ti,ab) |

Supplementary Table 2: Calculated statistics for Retinal sensitivity changes:

| **Subgroup within study** | **Group by Subgroup** | **Study Name** | **Point** | **Study Variance** | **Tau² Within** | **Tau² Between** | **Total Variance** | **IV-Weight** | **W** | **T×W** | **T²×W** | **W²** | **W³** | **C** | **Q** | **Q df** | **I²** | **B** | **K** | **Summary Point** | **Summary Variance** | **Group T²** | **Group T² Variance** |
| --- | --- | --- | --- | --- | --- | --- | --- | --- | --- | --- | --- | --- | --- | --- | --- | --- | --- | --- | --- | --- | --- | --- | --- |
| 12 months | 12 months | Fischer et al 2020 | 0.76 | 2.977 | 0 | 0 | 2.977 | 0.33590863 | 0.33590863 | 0.25529056 | 0.19402083 | 0.11283461 | 3.79E-02 | 0.43948713 | 8.79E-05 | 1 | 0 | 1 | 2 | 0.77308353 | 1.02951626 | 0 | 10.3547034 |
| 12 months | 12 months | MacLaren et al 2024 | 0.78 | 1.57375893 | 0 | 0 | 1.57375893 | 0.63542134 | 0.63542134 | 0.49562864 | 0.38659034 | 0.40376027 | 0.25655789 | 0.43948713 | 8.79E-05 | 1 | 0 | 1 | 2 | 0.77308353 | 1.02951626 | 0 | 10.3547034 |
| 12 months |  |  | 1.54 | 4.55075893 | 0 | 0 | 4.55075893 |  | 0.97132997 | 0.7509192 | 0.58061117 | 0.51659488 | 0.29446001 | 0.43948713 | 8.79E-05 | 1 | 0 | 1 | 2 | 0.77308353 | 1.02951626 | 0 | 10.3547034 |
| 24 months | 24 months | Aleman et al 2022 | 0.956 | 0.11882353 | 0 | 0 | 0.11882353 | 8.41584158 | 8.41584158 | 8.04554455 | 7.69154059 | 70.8263896 | 596.063675 | 16.6269303 | 0.19831742 | 3 | 0 | 3 | 4 | 0.81623086 | 1.35E-02 | 0 | 2.17E-02 |
| 24 months | 24 months | Fischer et al 2019 | 0.6 | 9.04333333 | 0 | 0 | 9.04333333 | 0.1105787 | 0.1105787 | 6.63E-02 | 0.03980833 | 1.22E-02 | 1.35E-03 | 16.6269303 | 0.19831742 | 3 | 0 | 3 | 4 | 0.81623086 | 1.35E-02 | 0 | 2.17E-02 |
| 24 months | 24 months | Lam et al 2018 | 0.7 | 1.15809083 | 0 | 0 | 1.15809083 | 0.86349013 | 0.86349013 | 0.60444309 | 0.42311016 | 0.7456152 | 0.64383136 | 16.6269303 | 0.19831742 | 3 | 0 | 3 | 4 | 0.81623086 | 1.35E-02 | 0 | 2.17E-02 |
| 24 months | 24 months | Xue et al 2018 | 0.8 | 1.54E-02 | 0 | 0 | 1.54E-02 | 64.8148148 | 64.8148148 | 51.8518519 | 41.4814815 | 4200.96022 | 272284.459 | 16.6269303 | 0.19831742 | 3 | 0 | 3 | 4 | 0.81623086 | 1.35E-02 | 0 | 2.17E-02 |
| 24 months |  |  | 3.056 | 10.3356763 | 0 | 0 | 10.3356763 |  | 74.2047252 | 60.5681867 | 49.6359406 | 4272.54445 | 272881.168 | 16.6269303 | 0.19831742 | 3 | 0 | 3 | 4 | 0.81623086 | 1.35E-02 | 0 | 2.17E-02 |
| 48 months | 48 months | Zhai et al 2022 | 0.93 | 0.84584 | 0 | 0 | 0.84584 | 1.18225669 | 1.18225669 | 1.09949872 | 1.02253381 | 1.39773088 | 1.65247669 | -1.33E-15 | 3.33E-15 | 0 | 0 | 0 | 1 | 0.93 | 0.84584 | 0 | 0 |
| 48 months |  |  | 0.93 | 0.84584 | 0 | 0 | 0.84584 |  | 1.18225669 | 1.09949872 | 1.02253381 | 1.39773088 | 1.65247669 | -1.33E-15 | 3.33E-15 | 0 | 0 | 0 | 1 | 0.93 | 0.84584 | 0 | 0 |
| 6 months | 6 months | MacLaren et al 2014 | 0.65 | 5.43E-02 | 0 | 0 | 5.43E-02 | 18.404908 | 18.404908 | 11.9631902 | 7.77607362 | 338.740638 | 6234.49026 | 1.19827448 | 4.85E-03 | 1 | 0 | 1 | 2 | 0.65292978 | 5.26E-02 | 0 | 1.39289177 |
| 6 months | 6 months | MacLaren et al 2024 | 0.74 | 1.61473333 | 0 | 0 | 1.61473333 | 0.6192973 | 0.6192973 | 0.45828 | 0.3391272 | 0.38352915 | 0.23751857 | 1.19827448 | 4.85E-03 | 1 | 0 | 1 | 2 | 0.65292978 | 5.26E-02 | 0 | 1.39289177 |
| 6 months |  |  | 1.39 | 1.66906667 | 0 | 0 | 1.66906667 |  | 19.0242053 | 12.4214702 | 8.11520082 | 339.124167 | 6234.72778 | 1.19827448 | 4.85E-03 | 1 | 0 | 1 | 2 | 0.65292978 | 5.26E-02 | 0 | 1.39289177 |
| Overall |  |  | 6.916 | 17.4013419 | 0 | 0 | 17.4013419 | 95.3825172 | 95.3825172 | 74.8400748 | 59.3542864 | 4613.58294 | 279117.842 | 47.0132449 | 0.63244754 | 8 | 0 | 8 | 9 | 0.78463095 | 1.05E-02 | 0 | 7.24E-03 |

Supplementary Table 3: Calculated statistics for Visual Acuity:

| **Subgroup within study** | **Group by Subgroup** | **Study Name** | **Point** | **Study Variance** | **Tau² Within** | **Tau² Between** | **Total Variance** | **IV-Weight** | **W** | **T×W** | **T²×W** | **W²** | **W³** | **C** | **Q** | **Q df** | **I²** | **B** | **K** | **Summary Point** | **Summary Variance** | **Group T²** | **Group T² Variance** |
| --- | --- | --- | --- | --- | --- | --- | --- | --- | --- | --- | --- | --- | --- | --- | --- | --- | --- | --- | --- | --- | --- | --- | --- |
| 12 months | 12 months | Lam et al 2018 | 2.2 | 5.08166667 | 0 | 0 | 5.08166667 | 0.19678583 | 0.19678583 | 0.43292883 | 0.95244342 | 0.03872466 | 7.62E-03 | 0.11518526 | 0.20790939 | 1 | 0 | 1 | 2 | 1.64393358 | 3.59443287 | 0 | 150.742672 |
| 12 months | 12 months | Fischer et al 2019 | 0.3 | 12.2816667 | 0 | 0 | 12.2816667 | 8.14E-02 | 8.14E-02 | 2.44E-02 | 7.33E-03 | 6.63E-03 | 5.40E-04 | 0.11518526 | 0.20790939 | 1 | 0 | 1 | 2 | 1.64393358 | 3.59443287 | 0 | 150.742672 |
| 12 months |  |  | 2.5 | 17.3633333 | 0 | 0 | 17.3633333 |  | 0.27820801 | 0.45735548 | 0.95977142 | 4.54E-02 | 8.16E-03 | 0.11518526 | 0.20790939 | 1 | 0 | 1 | 2 | 1.64393358 | 3.59443287 | 0 | 150.742672 |
| 18 months | 18 months | Lam et al 2018 | 4.2 | 7.44666667 | 0 | 0 | 7.44666667 | 0.13428827 | 0.13428827 | 0.56401074 | 2.36884512 | 1.80E-02 | 2.42E-03 | 7.45E-02 | 3.02E-02 | 1 | 0 | 1 | 2 | 3.95043753 | 5.38176834 | 0 | 360.595512 |
| 18 months | 18 months | Fischer et al 2019 | 3.3 | 19.4083333 | 0 | 0 | 19.4083333 | 5.15E-02 | 5.15E-02 | 0.17003006 | 0.56109918 | 2.65E-03 | 1.37E-04 | 7.45E-02 | 3.02E-02 | 1 | 0 | 1 | 2 | 3.95043753 | 5.38176834 | 0 | 360.595512 |
| 18 months |  |  | 7.5 | 26.855 | 0 | 0 | 26.855 |  | 0.18581253 | 0.7340408 | 2.92994431 | 2.07E-02 | 2.56E-03 | 7.45E-02 | 3.02E-02 | 1 | 0 | 1 | 2 | 3.95043753 | 5.38176834 | 0 | 360.595512 |
| 24 months | 24 months | Aleman et al 2022 | 0.2 | 13.6241667 | 0.93729554 | 0 | 14.5614622 | 6.87E-02 | 6.87E-02 | 1.37E-02 | 2.75E-03 | 4.72E-03 | 3.24E-04 | 0.44067316 | 2.7953585 | 3 | 0 | 3 | 4 | 4.07990319 | 1.46714106 | 0.93729554 | 27.9606579 |
| 24 months | 24 months | Lam et al 2018 | 2.5 | 3.70833333 | 0.93729554 | 0 | 4.64562887 | 0.21525611 | 0.21525611 | 0.53814028 | 1.34535069 | 0.04633519 | 9.97E-03 | 0.44067316 | 2.7953585 | 3 | 0 | 3 | 4 | 4.07990319 | 1.46714106 | 0.93729554 | 27.9606579 |
| 24 months | 24 months | Xue et al 2018 | 6 | 2.09857143 | 0.93729554 | 0 | 3.03586697 | 0.3293952 | 0.3293952 | 1.97637119 | 11.8582271 | 0.1085012 | 0.03573977 | 0.44067316 | 2.7953585 | 3 | 0 | 3 | 4 | 4.07990319 | 1.46714106 | 0.93729554 | 27.9606579 |
| 24 months | 24 months | Fischer et al 2019 | 3.7 | 13.71 | 0.93729554 | 0 | 14.6472955 | 6.83E-02 | 6.83E-02 | 0.25260636 | 0.93464353 | 4.66E-03 | 3.18E-04 | 0.44067316 | 2.7953585 | 3 | 0 | 3 | 4 | 4.07990319 | 1.46714106 | 0.93729554 | 27.9606579 |
| 24 months |  |  | 12.4 | 33.1410714 | 3.74918216 | 0 | 36.8902536 |  | 0.68159772 | 2.7808527 | 14.1409683 | 0.16421363 | 4.64E-02 | 0.44067316 | 2.7953585 | 3 | 0 | 3 | 4 | 4.07990319 | 1.46714106 | 0.93729554 | 27.9606579 |
| 3 months | 3 months | Lam et al 2018 | 0.2 | 5.92333333 | 0 | 0 | 5.92333333 | 0.16882386 | 0.16882386 | 3.38E-02 | 6.75E-03 | 2.85E-02 | 4.81E-03 | 5.57E-02 | 0.3607683 | 1 | 0 | 1 | 2 | 0.79359748 | 4.94664563 | 0 | 645.242939 |
| 3 months | 3 months | Fischer et al 2019 | 3.8 | 30 | 0 | 0 | 30 | 3.33E-02 | 3.33E-02 | 0.12666667 | 0.48133333 | 1.11E-03 | 3.70E-05 | 5.57E-02 | 0.3607683 | 1 | 0 | 1 | 2 | 0.79359748 | 4.94664563 | 0 | 645.242939 |
| 3 months |  |  | 4 | 35.9233333 | 0 | 0 | 35.9233333 |  | 0.20215719 | 0.16043144 | 0.48808629 | 2.96E-02 | 4.85E-03 | 5.57E-02 | 0.3607683 | 1 | 0 | 1 | 2 | 0.79359748 | 4.94664563 | 0 | 645.242939 |
| 54 months | 54 months | Zhai et al 2022 | 3 | 28.9917 | 0 | 0 | 28.9917 | 3.45E-02 | 3.45E-02 | 0.10347789 | 0.31043368 | 1.19E-03 | 4.10E-05 | 1.39E-16 | 2.22E-16 | 0 | 0 | 0 | 1 | 3 | 28.9917 | 0 | 0 |
| 54 months |  |  | 3 | 28.9917 | 0 | 0 | 28.9917 |  | 3.45E-02 | 0.10347789 | 0.31043368 | 1.19E-03 | 4.10E-05 | 1.39E-16 | 2.22E-16 | 0 | 0 | 0 | 1 | 3 | 28.9917 | 0 | 0 |
| 6 months | 6 months | Lam et al 2018 | 1.8 | 8.96666667 | 0 | 0 | 8.96666667 | 0.11152416 | 0.11152416 | 0.20074349 | 0.36133829 | 1.24E-02 | 1.39E-03 | 0.55761042 | 1.74325823 | 3 | 0 | 3 | 4 | 3.76728929 | 1.20495643 | 0 | 19.2969865 |
| 6 months | 6 months | MacLaren et al 2014 | 5.3 | 3.53666667 | 0 | 0 | 3.53666667 | 0.28275212 | 0.28275212 | 1.49858624 | 7.94250707 | 7.99E-02 | 2.26E-02 | 0.55761042 | 1.74325823 | 3 | 0 | 3 | 4 | 3.76728929 | 1.20495643 | 0 | 19.2969865 |
| 6 months | 6 months | Fischer et al 2019 | 0.9 | 12.7066667 | 0 | 0 | 12.7066667 | 7.87E-02 | 7.87E-02 | 7.08E-02 | 6.37E-02 | 6.19E-03 | 4.87E-04 | 0.55761042 | 1.74325823 | 3 | 0 | 3 | 4 | 3.76728929 | 1.20495643 | 0 | 19.2969865 |
| 6 months | 6 months | MacLaren 2014 | 3.8 | 2.80166667 | 0 | 0 | 2.80166667 | 0.3569304 | 0.3569304 | 1.35633551 | 5.15407496 | 0.12739931 | 4.55E-02 | 0.55761042 | 1.74325823 | 3 | 0 | 3 | 4 | 3.76728929 | 1.20495643 | 0 | 19.2969865 |
| 6 months |  |  | 11.8 | 28.0116667 | 0 | 0 | 28.0116667 |  | 0.82990553 | 3.12649421 | 13.5216664 | 0.22597922 | 7.00E-02 | 0.55761042 | 1.74325823 | 3 | 0 | 3 | 4 | 3.76728929 | 1.20495643 | 0 | 19.2969865 |
| 9 months | 9 months | Lam et al 2018 | 0.5 | 4.22166667 | 0 | 0 | 4.22166667 | 0.23687327 | 0.23687327 | 0.11843664 | 0.05921832 | 5.61E-02 | 1.33E-02 | 0.14314684 | 0.6441608 | 1 | 0 | 1 | 2 | 1.40647739 | 2.94605153 | 0 | 97.6037347 |
| 9 months | 9 months | Fischer et al 2019 | 3.5 | 9.75 | 0 | 0 | 9.75 | 0.1025641 | 0.1025641 | 0.35897436 | 1.25641026 | 1.05E-02 | 1.08E-03 | 0.14314684 | 0.6441608 | 1 | 0 | 1 | 2 | 1.40647739 | 2.94605153 | 0 | 97.6037347 |
| 9 months |  |  | 4 | 13.9716667 | 0 | 0 | 13.9716667 |  | 0.33943738 | 0.477411 | 1.31562857 | 6.66E-02 | 1.44E-02 | 0.14314684 | 0.6441608 | 1 | 0 | 1 | 2 | 1.40647739 | 2.94605153 | 0 | 97.6037347 |
| Overall |  |  | 45.2 | 184.257771 | 3.74918216 | 0 | 188.006954 | 2.55161098 | 2.55161098 | 7.84006352 | 33.6664989 | 0.55366586 | 0.14628683 | 2.3346242 | 9.57717013 | 16 | 0 | 16 | 17 | 3.07259358 | 0.39190927 | 0 | 5.08269183 |

Supplementary Table 4: Calculated statistics for Preserved Retinal Pigmented Epithelium Area (Fundus Autofluorescence)

| **Subgroup within study** | **Group by Subgroup** | **Study Name** | **Point** | **Study Variance** | **Tau² Within** | **Tau² Between** | **Total Variance** | **IV-Weight** | **W** | **T×W** | **T²×W** | **W²** | **W³** | **C** | **Q** | **Q df** | **I²** | **B** | **K** | **Summary Point** | **Summary Variance** | **Group T²** | **Group T² Variance** |
| --- | --- | --- | --- | --- | --- | --- | --- | --- | --- | --- | --- | --- | --- | --- | --- | --- | --- | --- | --- | --- | --- | --- | --- |
| 12 months | 12 months | Dimopoulos et al 2018 | -5.56 | 10.84192 | 0 | 0 | 10.84192 | 9.22E-02 | 9.22E-02 | -0.5128243 | 2.85130309 | 8.51E-03 | 7.85E-04 | 2.25E-02 | 3.53E-03 | 1 | 0 | 1 | 2 | -5.4916598 | 9.51881454 | 0 | 3946.44337 |
| 12 months | 12 months | Fischer et al 2020 | -5 | 78 | 0 | 0 | 78 | 1.28E-02 | 1.28E-02 | -0.0641026 | 0.32051282 | 1.64E-04 | 2.11E-06 | 2.25E-02 | 3.53E-03 | 1 | 0 | 1 | 2 | -5.4916598 | 9.51881454 | 0 | 3946.44337 |
| 12 months |  |  | -10.56 | 88.84192 | 0 | 0 | 88.84192 |  | 0.1050551 | -0.5769269 | 3.17181591 | 8.67E-03 | 7.87E-04 | 2.25E-02 | 3.53E-03 | 1 | 0 | 1 | 2 | -5.4916598 | 9.51881454 | 0 | 3946.44337 |
| 24 months | 24 months | Dimopoulos et al 2018 | -4.83 | 15.92818 | 0 | 0 | 15.92818 | 6.28E-02 | 6.28E-02 | -0.3032362 | 1.46463061 | 3.94E-03 | 2.47E-04 | 0.11642677 | 4.01E-02 | 1 | 0 | 1 | 2 | -4.0603964 | 1.15904159 | 0 | 147.544934 |
| 24 months | 24 months | Fischer et al 2019 | -4 | 1.25 | 0 | 0 | 1.25 | 0.8 | 0.8 | -3.2 | 12.8 | 0.64 | 0.512 | 0.11642677 | 4.01E-02 | 1 | 0 | 1 | 2 | -4.0603964 | 1.15904159 | 0 | 147.544934 |
| 24 months |  |  | -8.83 | 17.17818 | 0 | 0 | 17.17818 |  | 0.86278181 | -3.5032362 | 14.2646306 | 0.64394156 | 0.51224746 | 0.11642677 | 4.01E-02 | 1 | 0 | 1 | 2 | -4.0603964 | 1.15904159 | 0 | 147.544934 |
| 54 months | 54 months | Zhai et al 2022 | -16 | 61.2 | 0 | 0 | 61.2 | 1.63E-02 | 1.63E-02 | -0.2614379 | 4.18300654 | 2.67E-04 | 4.36E-06 | 1.39E-17 | 0 | 0 | 0 | 0 | 1 | -16 | 61.2 | 0 | 0 |
| 54 months |  |  | -16 | 61.2 | 0 | 0 | 61.2 |  | 1.63E-02 | -0.2614379 | 4.18300654 | 2.67E-04 | 4.36E-06 | 1.39E-17 | 0 | 0 | 0 | 0 | 1 | -16 | 61.2 | 0 | 0 |
| Overall |  |  | -35.39 | 167.2201 | 0 | 0 | 167.2201 | 0.98417678 | 0.98417678 | -4.3416009 | 21.6194531 | 0.65288013 | 0.51303859 | 0.32079989 | 2.46689943 | 4 | 0 | 4 | 5 | -4.4114035 | 1.01607762 | 0 | 77.7358885 |

Supplementary Table 5: Calculated statistics for TRAE

| **Subgroup within study** | **Group by Subgroup** | **Study Name** | **Point** | **Study Variance** | **Tau² Within** | **Tau² Between** | **Total Variance** | **IV-Weight** | **W** | **T×W** | **T²×W** | **W²** | **W³** | **C** | **Q** | **Q df** | **I²** | **B** | **K** | **Summary Point** | **Summary Variance** | **Group T²** | **Group T² Variance** |
| --- | --- | --- | --- | --- | --- | --- | --- | --- | --- | --- | --- | --- | --- | --- | --- | --- | --- | --- | --- | --- | --- | --- | --- |
| Anemia | Anemia | Dimopoulos et al 2018 | -1.6094379 | 1.2 | 0 | 0 | 1.2 | 0.83333333 | 0.83333333 | -1.3411983 | 2.15857533 | 0.69444444 | 0.5787037 | 0 | 0 | 0 | 0 | 0 | 1 | -1.6094379 | 1.2 | 0 | 0 |
|  | Anemia |  | -1.6094379 | 1.2 | 0 | 0 | 1.2 |  | 0.83333333 | -1.3411983 | 2.15857533 | 0.69444444 | 0.5787037 | 0 | 0 | 0 | 0 | 0 | 1 | -1.6094379 | 1.2 | 0 | 0 |
| Any TRAE | Any TRAE | Dimopoulos et al 2018 | 2.56494936 | 2.15384615 | 12.7368446 | 0 | 14.8906908 | 6.72E-02 | 6.72E-02 | 0.17225187 | 0.44181733 | 4.51E-03 | 3.03E-04 | 0.13982294 | 1.61502334 | 2 | 0 | 2 | 3 | 1.72753231 | 4.76165009 | 12.7368446 | 216.863659 |
| Any TRAE | Any TRAE | Xue et al 2018 | -1.7917595 | 0.58333333 | 12.7368446 | 0 | 13.320178 | 7.51E-02 | 7.51E-02 | -0.1345147 | 0.24101795 | 5.64E-03 | 4.23E-04 | 0.13982294 | 1.61502334 | 2 | 0 | 2 | 3 | 1.72753231 | 4.76165009 | 12.7368446 | 216.863659 |
| Any TRAE | Any TRAE | MacLaren et al 2024 | 4.79579055 | 2.01652893 | 12.7368446 | 0 | 14.7533735 | 6.78E-02 | 6.78E-02 | 0.325064 | 1.55893883 | 4.59E-03 | 0.00031141 | 0.13982294 | 1.61502334 | 2 | 0 | 2 | 3 | 1.72753231 | 4.76165009 | 12.7368446 | 216.863659 |
|  | Any TRAE |  | 5.56898043 | 4.75370841 | 38.2105339 | 0 | 42.9642423 |  | 0.21001123 | 0.36280119 | 2.24177411 | 1.47E-02 | 1.04E-03 | 0.13982294 | 1.61502334 | 2 | 0 | 2 | 3 | 1.72753231 | 4.76165009 | 12.7368446 | 216.863659 |
| Cataract | Cataract | Lam et al 2018 | -1.6094379 | 1.2 | 0 | 0 | 1.2 | 0.83333333 | 0.83333333 | -1.3411983 | 2.15857533 | 0.69444444 | 0.5787037 | 0 | 0 | 0 | 0 | 0 | 1 | -1.6094379 | 1.2 | 0 | 0 |
|  | Cataract |  | -1.6094379 | 1.2 | 0 | 0 | 1.2 |  | 0.83333333 | -1.3411983 | 2.15857533 | 0.69444444 | 0.5787037 | 0 | 0 | 0 | 0 | 0 | 1 | -1.6094379 | 1.2 | 0 | 0 |
| Conjunctiva hemorrhage | Conjunctiva hemorrhage | Lam et al 2018 | 2.56494936 | 2.15384615 | 0 | 0 | 2.15384615 | 0.46428571 | 0.46428571 | 1.19086934 | 3.05451956 | 0.21556122 | 0.100082 | -5.55E-16 | 1.02E-14 | 0 | 0 | 0 | 1 | 2.56494936 | 2.15384615 | 0 | 0 |
|  | Conjunctiva hemorrhage | 2.56494936 | 2.15384615 | 0 | 0 | 2.15384615 |  | 0.46428571 | 1.19086934 | 3.05451956 | 0.21556122 | 0.100082 | -5.55E-16 | 1.02E-14 | 0 | 0 | 0 | 1 | 2.56494936 | 2.15384615 | 0 | 0 |  |
| Epigastric pain | Epigastric pain | Dimopoulos et al 2018 | -1.6094379 | 1.2 | 0 | 0 | 1.2 | 0.83333333 | 0.83333333 | -1.3411983 | 2.15857533 | 0.69444444 | 0.5787037 | 0 | 0 | 0 | 0 | 0 | 1 | -1.6094379 | 1.2 | 0 | 0 |
|  | Epigastric pain | -1.6094379 | 1.2 | 0 | 0 | 1.2 |  | 0.83333333 | -1.3411983 | 2.15857533 | 0.69444444 | 0.5787037 | 0 | 0 | 0 | 0 | 0 | 1 | -1.6094379 | 1.2 | 0 | 0 |  |
| foveal thinning | foveal thinning | Aleman et al 2022 | -2.014903 | 0.56666667 | 0 | 0 | 0.56666667 | 1.76470588 | 1.76470588 | -3.5557112 | 7.16441326 | 3.11418685 | 5.49562386 | -8.88E-16 | 1.24E-14 | 0 | 0 | 0 | 1 | -2.014903 | 0.56666667 | 0 | 0 |
|  | foveal thinning | -2.014903 | 0.56666667 | 0 | 0 | 0.56666667 |  | 1.76470588 | -3.5557112 | 7.16441326 | 3.11418685 | 5.49562386 | -8.88E-16 | 1.24E-14 | 0 | 0 | 0 | 1 | -2.014903 | 0.56666667 | 0 | 0 |  |
| inflammation | inflammation | Xue et al 2018 | -3.1354942 | 1.04347826 | 0 | 0 | 1.04347826 | 0.95833333 | 0.95833333 | -3.0048486 | 9.42168548 | 0.91840278 | 0.880136 | 5.55E-16 | 0 | 0 | 0 | 0 | 1 | -3.1354942 | 1.04347826 | 0 | 0 |
|  | inflammation | -3.1354942 | 1.04347826 | 0 | 0 | 1.04347826 |  | 0.95833333 | -3.0048486 | 9.42168548 | 0.91840278 | 0.880136 | 5.55E-16 | 0 | 0 | 0 | 0 | 1 | -3.1354942 | 1.04347826 | 0 | 0 |  |
| IOP decrease | IOP decrease | Dimopoulos et al 2018 | 2.56494936 | 2.15384615 | 0 | 0 | 2.15384615 | 0.46428571 | 0.46428571 | 1.19086934 | 3.05451956 | 0.21556122 | 0.100082 | -5.55E-16 | 1.02E-14 | 0 | 0 | 0 | 1 | 2.56494936 | 2.15384615 | 0 | 0 |
|  | IOP decrease | 2.56494936 | 2.15384615 | 0 | 0 | 2.15384615 |  | 0.46428571 | 1.19086934 | 3.05451956 | 0.21556122 | 0.100082 | -5.55E-16 | 1.02E-14 | 0 | 0 | 0 | 1 | 2.56494936 | 2.15384615 | 0 | 0 |  |
| macular retinal hole | macular retinal hole | Lam et al 2018 | -0.6931472 | 0.75 | 0 | 0 | 0.75 | 1.33333333 | 1.33333333 | -0.9241962 | 0.64060402 | 1.77777778 | 2.37037037 | -8.88E-16 | 2.22E-16 | 0 | 0 | 0 | 1 | -0.6931472 | 0.75 | 0 | 0 |
|  | macular retinal hole | -0.6931472 | 0.75 | 0 | 0 | 0.75 |  | 1.33333333 | -0.9241962 | 0.64060402 | 1.77777778 | 2.37037037 | -8.88E-16 | 2.22E-16 | 0 | 0 | 0 | 1 | -0.6931472 | 0.75 | 0 | 0 |  |
| Metamorphopsia | Metamorphopsia | Dimopoulos et al 2018 | 2.56494936 | 2.15384615 | 0 | 0 | 2.15384615 | 0.46428571 | 0.46428571 | 1.19086934 | 3.05451956 | 0.21556122 | 0.100082 | -5.55E-16 | 1.02E-14 | 0 | 0 | 0 | 1 | 2.56494936 | 2.15384615 | 0 | 0 |
|  | Metamorphopsia | 2.56494936 | 2.15384615 | 0 | 0 | 2.15384615 |  | 0.46428571 | 1.19086934 | 3.05451956 | 0.21556122 | 0.100082 | -5.55E-16 | 1.02E-14 | 0 | 0 | 0 | 1 | 2.56494936 | 2.15384615 | 0 | 0 |  |
| Occular pain | Occular pain | Dimopoulos et al 2018 | 0.69314718 | 0.75 | 0 | 0 | 0.75 | 1.33333333 | 1.33333333 | 0.92419624 | 0.64060402 | 1.77777778 | 2.37037037 | -8.88E-16 | 2.22E-16 | 0 | 0 | 0 | 1 | 0.69314718 | 0.75 | 0 | 0 |
|  | Occular pain | 0.69314718 | 0.75 | 0 | 0 | 0.75 |  | 1.33333333 | 0.92419624 | 0.64060402 | 1.77777778 | 2.37037037 | -8.88E-16 | 2.22E-16 | 0 | 0 | 0 | 1 | 0.69314718 | 0.75 | 0 | 0 |  |
| Occular TRAE | Occular TRAE | Dimopoulos et al 2018 | 2.56494936 | 2.15384615 | 4.23453872 | 0 | 6.38838488 | 0.15653409 | 0.15653409 | 0.40150201 | 1.02983232 | 2.45E-02 | 3.84E-03 | 0.59613524 | 2.84537476 | 3 | 0 | 3 | 4 | 1.02876712 | 1.25137843 | 4.23453872 | 18.4604549 |
| Occular TRAE | Occular TRAE | Xue et al 2018 | -1.7917595 | 0.58333333 | 4.23453872 | 0 | 4.81787206 | 0.20756051 | 0.20756051 | -0.3718985 | 0.66635269 | 4.31E-02 | 8.94E-03 | 0.59613524 | 2.84537476 | 3 | 0 | 3 | 4 | 1.02876712 | 1.25137843 | 4.23453872 | 18.4604549 |
| Occular TRAE | Occular TRAE | MacLaren et al 2024 | 2.94443898 | 0.35087719 | 4.23453872 | 0 | 4.58541592 | 0.21808273 | 0.21808273 | 0.64213128 | 1.89071636 | 4.76E-02 | 1.04E-02 | 0.59613524 | 2.84537476 | 3 | 0 | 3 | 4 | 1.02876712 | 1.25137843 | 4.23453872 | 18.4604549 |
| Occular TRAE | Occular TRAE | Fischer et al 2020 | 0.69314718 | 0.375 | 4.23453872 | 0 | 4.60953872 | 0.21694145 | 0.21694145 | 0.15037235 | 0.10423017 | 4.71E-02 | 1.02E-02 | 0.59613524 | 2.84537476 | 3 | 0 | 3 | 4 | 1.02876712 | 1.25137843 | 4.23453872 | 18.4604549 |
|  | Occular TRAE | 4.41077605 | 3.46305668 | 16.9381549 | 0 | 20.4012116 |  | 0.79911877 | 0.82210712 | 3.69113154 | 0.16220795 | 3.34E-02 | 0.59613524 | 2.84537476 | 3 | 0 | 3 | 4 | 1.02876712 | 1.25137843 | 4.23453872 | 18.4604549 |  |
| Ocular SAE | Ocular SAE | Dimopoulos et al 2018 | -1.6094379 | 1.2 | 1.01281568 | 0 | 2.21281568 | 0.45191292 | 0.45191292 | -0.7273258 | 1.1705857 | 0.20422529 | 9.23E-02 | 1.63717698 | 5.02691728 | 3 | 40.3212778 | 8.55355541 | 4 | -1.101107 | 0.43660568 | 1.01281568 | 2.09274529 |
| Ocular SAE | Ocular SAE | Aleman et al 2022 | -2.014903 | 0.56666667 | 1.01281568 | 0 | 1.57948235 | 0.63311882 | 0.63311882 | -1.275673 | 2.57035742 | 0.40083944 | 0.25377899 | 1.63717698 | 5.02691728 | 3 | 40.3212778 | 8.55355541 | 4 | -1.101107 | 0.43660568 | 1.01281568 | 2.09274529 |
| Ocular SAE | Ocular SAE | MacLaren et al 2024 | -1.493925 | 0.11131725 | 1.01281568 | 0 | 1.12413294 | 0.8895745 | 0.8895745 | -1.3289576 | 1.98536303 | 0.7913428 | 0.70395837 | 1.63717698 | 5.02691728 | 3 | 40.3212778 | 8.55355541 | 4 | -1.101107 | 0.43660568 | 1.01281568 | 2.09274529 |
| Ocular SAE | Ocular SAE | Lam et al 2018 | 2.56494936 | 2.15384615 | 1.01281568 | 0 | 3.16666184 | 0.31578996 | 0.31578996 | 0.80998524 | 2.07757113 | 9.97E-02 | 3.15E-02 | 1.63717698 | 5.02691728 | 3 | 40.3212778 | 8.55355541 | 4 | -1.101107 | 0.43660568 | 1.01281568 | 2.09274529 |
|  | Ocular SAE |  | -2.5533166 | 4.03183007 | 4.05126274 | 0 | 8.08309281 |  | 2.2903962 | -2.5219712 | 7.80387728 | 1.49613082 | 1.08152103 | 1.63717698 | 5.02691728 | 3 | 40.3212778 | 8.55355541 | 4 | -1.101107 | 0.43660568 | 1.01281568 | 2.09274529 |
| retinal stretch | retinal stretch | Xue et al 2018 | -2.5649494 | 1.07692308 | 0 | 0 | 1.07692308 | 0.92857143 | 0.92857143 | -2.3817387 | 6.10903912 | 0.8622449 | 0.80065598 | 0 | 2.13E-14 | 0 | 0 | 0 | 1 | -2.5649494 | 1.07692308 | 0 | 0 |
|  | retinal stretch | -2.5649494 | 1.07692308 | 0 | 0 | 1.07692308 |  | 0.92857143 | -2.3817387 | 6.10903912 | 0.8622449 | 0.80065598 | 0 | 2.13E-14 | 0 | 0 | 0 | 1 | -2.5649494 | 1.07692308 | 0 | 0 |  |
| Subconjunctival hemorrhage | Subconjunctival hemorrhage | Dimopoulos et al 2018 | 2.56494936 | 2.15384615 | 0 | 0 | 2.15384615 | 0.46428571 | 0.46428571 | 1.19086934 | 3.05451956 | 0.21556122 | 0.100082 | -5.55E-16 | 1.02E-14 | 0 | 0 | 0 | 1 | 2.56494936 | 2.15384615 | 0 | 0 |
|  | Subconjunctival hemorrhage | 2.56494936 | 2.15384615 | 0 | 0 | 2.15384615 |  | 0.46428571 | 1.19086934 | 3.05451956 | 0.21556122 | 0.100082 | -5.55E-16 | 1.02E-14 | 0 | 0 | 0 | 1 | 2.56494936 | 2.15384615 | 0 | 0 |  |
| Subretinal fluid | Subretinal fluid | Lam et al 2018 | 1.60943791 | 1.2 | 0 | 0 | 1.2 | 0.83333333 | 0.83333333 | 1.34119826 | 2.15857533 | 0.69444444 | 0.5787037 | 0 | 0 | 0 | 0 | 0 | 1 | 1.60943791 | 1.2 | 0 | 0 |
|  | Subretinal fluid | 1.60943791 | 1.2 | 0 | 0 | 1.2 |  | 0.83333333 | 1.34119826 | 2.15857533 | 0.69444444 | 0.5787037 | 0 | 0 | 0 | 0 | 0 | 1 | 1.60943791 | 1.2 | 0 | 0 |  |
| Suture inflammation | Suture inflammation | Dimopoulos et al 2018 | -1.6094379 | 1.2 | 0 | 0 | 1.2 | 0.83333333 | 0.83333333 | -1.3411983 | 2.15857533 | 0.69444444 | 0.5787037 | 0 | 0 | 0 | 0 | 0 | 1 | -1.6094379 | 1.2 | 0 | 0 |
|  | Suture inflammation | -1.6094379 | 1.2 | 0 | 0 | 1.2 |  | 0.83333333 | -1.3411983 | 2.15857533 | 0.69444444 | 0.5787037 | 0 | 0 | 0 | 0 | 0 | 1 | -1.6094379 | 1.2 | 0 | 0 |  |
| Vitreous floaters | Vitreous floaters | Dimopoulos et al 2018 | -0.6931472 | 0.75 | 0 | 0 | 0.75 | 1.33333333 | 1.33333333 | -0.9241962 | 0.64060402 | 1.77777778 | 2.37037037 | -8.88E-16 | 2.22E-16 | 0 | 0 | 0 | 1 | -0.6931472 | 0.75 | 0 | 0 |
|  | Vitreous floaters | -0.6931472 | 0.75 | 0 | 0 | 0.75 |  | 1.33333333 | -0.9241962 | 0.64060402 | 1.77777778 | 2.37037037 | -8.88E-16 | 2.22E-16 | 0 | 0 | 0 | 1 | -0.6931472 | 0.75 | 0 | 0 |  |
|  | Overall |  | 4.4494298 | 31.8010478 | 59.1999515 | 0 | 91.0009993 | 16.9749464 | 16.9749464 | -10.463675 | 61.3646877 | 16.2357141 | 18.6972915 | 16.0184948 | 54.9146824 | 25 | 54.4748346 | 136.96041 | 26 | -0.6164187 | 5.89E-02 | 3.09080997 | 1.99199326 |

Supplementary Table 6: Calculated statistics for Sub-foveal Choroidal thickness

| **Study name** | Point | **Study variance** | **Tau² Within** | **Tau² Between** | **Total Variance** | **IV-Weight** | **W** | **T×W** | **T²×W** | **W²** | **W³** | **C** | **Q** | **Q df** | **I²** | **B** | **K** | **Summary Point** | **Summary Variance** | **Group T²** | **Group T² Variance** |
| --- | --- | --- | --- | --- | --- | --- | --- | --- | --- | --- | --- | --- | --- | --- | --- | --- | --- | --- | --- | --- | --- |
| Fischer et al 2020 | 4.1 | 60 | 0 | 0 | 60 | 1.67E-02 | 1.67E-02 | 6.83E-02 | 0.28016667 | 2.78E-04 | 4.63E-06 | 0.8351555 | 3.79497334 | 4 | 0 | 4 | 5 | 9.12650779 | 0.66059203 | 0 | 11.4697854 |
| Fischer et al 2019 | 7 | 38.9366667 | 0 | 0 | 38.9366667 | 2.57E-02 | 2.57E-02 | 0.17977913 | 1.2584539 | 6.60E-04 | 1.69E-05 | 0.8351555 | 3.79497334 | 4 | 0 | 4 | 5 | 9.12650779 | 0.66059203 | 0 | 11.4697854 |
| MacLaren et al 2014 | 6 | 23.5252501 | 0 | 0 | 23.5252501 | 4.25E-02 | 4.25E-02 | 0.25504511 | 1.53027066 | 1.81E-03 | 7.68E-05 | 0.8351555 | 3.79497334 | 4 | 0 | 4 | 5 | 9.12650779 | 0.66059203 | 0 | 11.4697854 |
| Lam et al 2018 | 8 | 1.32073933 | 0 | 0 | 1.32073933 | 0.75715168 | 0.75715168 | 6.05721341 | 48.4577073 | 0.57327866 | 0.4340589 | 0.8351555 | 3.79497334 | 4 | 0 | 4 | 5 | 9.12650779 | 0.66059203 | 0 | 11.4697854 |
| Xu et al 2018 | 10.8 | 1.48857143 | 0 | 0 | 1.48857143 | 0.67178503 | 0.67178503 | 7.25527831 | 78.3570058 | 0.45129512 | 0.30317331 | 0.8351555 | 3.79497334 | 4 | 0 | 4 | 5 | 9.12650779 | 0.66059203 | 0 | 11.4697854 |
|  | 35.9 | 125.271228 | 0 | 0 | 125.271228 | 1.51379362 | 1.51379362 | 13.8156493 | 129.883604 | 1.02731806 | 0.73733058 | 0.8351555 | 3.79497334 | 4 | 0 | 4 | 5 | 9.12650779 | 0.66059203 | 0 | 11.4697854 |

Supplementary Table 7: Events of Adverse events reported in studies

| **Study** | **ADR Category** | **Events** | **total Population** |
| --- | --- | --- | --- |
| Dimopoulos et al 2018 | Anemia | 1 | 6 |
| Dimopoulos et al 2018 | Any TRAE | 6 | 6 |
| Dimopoulos et al 2018 | Epigastric pain | 1 | 6 |
| Dimopoulos et al 2018 | IOP decrease | 6 | 6 |
| Dimopoulos et al 2018 | Metamorphopsia | 6 | 6 |
| Dimopoulos et al 2018 | Occular pain | 4 | 6 |
| Dimopoulos et al 2018 | Occular TRAE | 6 | 6 |
| Dimopoulos et al 2018 | Ocular SAE | 1 | 6 |
| Dimopoulos et al 2018 | Subconjunctival hemorrhage | 6 | 6 |
| Dimopoulos et al 2018 | Suture inflammation | 1 | 6 |
| Dimopoulos et al 2018 | Vitreous floaters | 2 | 6 |
| Aleman et al 2022 | foveal thinning | 2 | 17 |
| Aleman et al 2022 | Ocular SAE | 2 | 17 |
| Xue et al 2018 | Any TRAE | 2 | 14 |
| Xue et al 2018 | inflammation | 1 | 24 |
| Xue et al 2018 | Occular TRAE | 2 | 14 |
| Xue et al 2018 | retinal stretch | 1 | 14 |
| MacLaren et al 2024 | Any TRAE | 60 | 60 |
| MacLaren et al 2024 | Occular TRAE | 57 | 60 |
| MacLaren et al 2024 | Ocular SAE | 11 | 60 |
| Fischer et al 2020 | Occular TRAE | 8 | 12 |
| Lam et al 2018 | Cataract | 1 | 6 |
| Lam et al 2018 | Conjunctiva hemorrhage | 6 | 6 |
| Lam et al 2018 | macular retinal hole | 2 | 6 |
| Lam et al 2018 | Ocular SAE | 6 | 6 |
| Lam et al 2018 | Subretinal fluid | 5 | 6 |
