# Supplementary material for: Efficacy and safety of AAV-mediated gene therapy for choroideremia: a systematic review and meta-analysis
Source: eClinicalMedicine. 2026 May 11;95:103923. doi: 10.1016/j.eclinm.2026.103923 (PMC13191274; doi:10.1016/j.eclinm.2026.103923)
Supplement: Translated Abstract [file mmc3.docx]

The following translations in Chinese were submitted by the authors and we reproduce them as supplied. They have not been peer reviewed. Our editorial processes have only been applied to the original abstract in English, which should serve as reference for this manuscript.

Translated abstract

摘要

背景

脈絡膜缺失症（choroideremia）是一種罕見的 X 聯遺傳性視網膜退化疾病，由 CHM 基因突變所致，會造成視網膜色素上皮、脈絡膜與光感受器的進行性退化。目前臨床處置仍以支持性治療為主，而基因治療已被視為具潛力的疾病修飾治療策略。本研究旨在系統性評估腺相關病毒（AAV）載體基因治療用於脈絡膜缺失症之療效與安全性。

方法

本系統性回顧與統合分析依循 PRISMA 2020 指引進行，並已於 PROSPERO 註冊（CRD420251146173）。研究團隊檢索 PubMed、Embase、Scopus、ScienceDirect、Web of Science 與 Cochrane Library 六個資料庫，自建庫起至 2025 年 12 月 3 日止。納入評估以 REP1 基因為標的之 AAV 載體基因治療的臨床試驗及前瞻性觀察研究。主要結局包含最佳矯正視力（BCVA）、以微視野檢查測得之視網膜敏感度、眼底自體螢光下保留之視網膜色素上皮（RPE）面積、黃斑中心下脈絡膜厚度，以及治療後新發不良事件（TEAEs）。統合分析採隨機效應模型，並使用 Comprehensive Meta-Analysis 軟體進行統計分析。

結果

本研究最終共納入 11 項臨床研究，合計 308 名受試者。AAV 載體基因治療可顯著提升視網膜敏感度，合併平均差（MD）為 0.78 dB（95% CI 0.58–0.99；p<0.001），且此效果於最長 48 個月追蹤期間內大致一致。BCVA 亦呈現顯著改善，合併 MD 為 3.07 個 ETDRS 字母（95% CI 1.85–4.30；p<0.001），其中以 24 個月追蹤時的改善較為明顯。結構性結果顯示，RPE 退化程度下降（MD -4.41，95% CI -6.39 至 -2.44；p<0.001），黃斑中心下脈絡膜厚度增加（MD 9.13 μm，95% CI 7.53–10.72；p<0.001）。在合併事件率分析中，約 35% 的治療眼出現 TEAEs，但多數為輕度至中度，且主要與手術操作相關；嚴重不良事件發生率相對較低（<20%）。

結論

AAV 載體基因治療對脈絡膜缺失症可帶來適度但具一致性的功能與結構效益。治療後新發不良事件並不少見，但多為與手術相關的眼部事件，且以輕度至中度為主，嚴重併發症相對少見。整體而言，此療法較可能作為延緩視網膜退化進程的疾病修飾治療，而非恢復既有視功能的治療方式；未來仍需進一步研究，以評估更早期介入、最佳化給藥與遞送策略，以及更長期追蹤的臨床價值。

資金來源

本研究未接受任何公部門、商業機構或非營利組織之特定經費補助。

關鍵詞

AAV2 載體基因治療；脈絡膜缺失症；CHM；REP1；微視野檢查；眼底自體螢光。
